# ZINBMM: a general mixture model for simultaneous clustering and gene selection using single-cell transcriptomic data

**DOI:** 10.1186/s13059-023-03046-0

**Published:** 2023-09-11

**Authors:** Yang Li, Mingcong Wu, Shuangge Ma, Mengyun Wu

**Affiliations:** 1 Center for Applied Statistics and School of Statistics, Renmin University of China, Beijing, China; 2 RSS and China-Re Life Joint Lab on Public Health and Risk Management, Renmin University of China, Beijing, China; 3 Statistical Consulting Center, Renmin University of China, Beijing, China; 4 Department of Biostatistics, Yale University, New Haven, USA; 5 School of Statistics and Management, Shanghai University of Finance and Economics, Shanghai, China

**Keywords:** Clustering analysis, Gene selection, ScRNA-seq data

## Abstract

Clustering is a critical component of single-cell RNA sequencing (scRNA-seq) data analysis and can help reveal cell types and infer cell lineages. Despite considerable successes, there are few methods tailored to investigating cluster-specific genes contributing to cell heterogeneity, which can promote biological understanding of cell heterogeneity. In this study, we propose a zero-inflated negative binomial mixture model (ZINBMM) that simultaneously achieves effective scRNA-seq data clustering and gene selection. ZINBMM conducts a systemic analysis on raw counts, accommodating both batch effects and dropout events. Simulations and the analysis of five scRNA-seq datasets demonstrate the practical applicability of ZINBMM.

**Supplementary Information:**

The online version contains supplementary material available at 10.1186/s13059-023-03046-0.

## Background

Single-cell RNA sequencing (scRNA-seq) has been widely conducted to determine heterogeneity and diversity of cell populations. It helps determine cellular differences with high specificity and provide a deeper understanding of complex biological variations at the individual cell level [1]. Most scRNA-seq studies involve clustering analysis to reveal cell types and infer cell lineages [2]. Available methods range from those based on classic K-means and spectral clustering [3, 4] to the more recent model-based clustering [5, 6]. We refer to [7] and [8] for more discussions.

Although scRNA-seq data clustering analysis is very promising owing to its extensive applications, several challenges exist because of technical and biological reasons, such as batch effects and dropout events. Systematic differences in gene expressions between batches caused by data generated at different times are known as batch effects. Several batch effect correction methods have been developed for scRNA-seq data, such as mutual nearest neighbours (MNN) [9] and canonical correlation analysis [10]. These methods have been extensively used in preprocessing technical batch effects in scRNA-seq clustering [11, 12]. Moreover, during cell-level resolution, the dropout phenomenon leads to a large amount of zero-count observations in gene expression data, which may result in a serious loss of information in scRNA-seq clustering. Many imputation methods have been proposed to recover dropouts, followed by a traditional clustering method, such as CIDR [13] and CMF-Impute [14]. A few other studies take a different strategy and adopt zero-inflated distributions to model zeros due to dropout, such as ZIFA [15] and scDeepCluster [5]. However, among these methods, few have considered both batch effects and dropout events simultaneously. In addition, batch effect removal and dropout imputation or modelling are typically addressed at the preprocessing stage before clustering. Such multi-staged strategies may lead to a loss of information and even introduce new bias across the various steps.

Another challenge is the high dimensionality of scRNA-seq data. This usually produces extensive noises, resulting in poor clustering accuracy and significantly adding to computation time. Dimension reduction is commonly conducted in scRNA-seq as a preliminary step prior to clustering. This step projects high-dimensional gene expression data into a lower dimension space and helps focus on relevant low-dimensional signals for better clustering [16]. Examples include incorporating principal component analysis into K-means clustering [17] and zero-inflated models [15, 18] and performing feature transformation through a stacked autoencoder [12]. However, the transformed lower-dimensional representations often suffer from an ineffective recover of useful information and a lack of biological interpretability [6, 18].

Feature selection can be used as an alternative to dimension reduction. It has attracted attention in recently published studies on scRNA-seq clustering and can output a subset of the original genes without transforming them. Because the majority of the genes are not differentially expressed among various cell types, feature selection can enhance signal-to-noise ratios and subsequently improve cell clustering by reducing the number of genes under consideration [19, 20]. Moreover, the selected cell type-specific genes may additionally assist in understanding biological cell types. Examples of such developments are FSCseq [21] and snbClust [22], which are two negative binomial (NB) mixture model-based methods and perform feature selection using the penalisation technique. However, these models do not consider dropouts and may be ineffective with highly sparse data. Another example is RZiMM [6], which simultaneously conducts clustering and feature scoring along with an adjustment for dropout events and batch heterogeneity. Opposed to the mixture model, RZiMM introduces binary subgroup indicators and conducts hard clustering, and it models all zeros with one component and ignores the differences between dropout zeros and expression zeros, which may lead to additional bias. In addition, it requires another threshold for selecting important genes, which cannot be determined automatically and may involve some subjectivity. Clustering analysis that can also conduct gene selection with effective adjustment of batch effects and dropouts is still limited for scRNA-seq data.

In this study, we propose a zero-inflated negative binomial mixture model (ZINBMM) for scRNA-seq data clustering that can comprehensively account for the unique problems of batch effects, dropout events, and high dimensionality. The model directly applies to the raw counts without any transformation to avoid a potential loss of information. The mixture model with biological effects of genes being modelled using cell type-specific mean parameters is developed to accommodate heterogeneity, which achieves soft clustering and has the advantage of more meaningful probabilistic interpretations. In addition to dealing with high sparsity owing to dropouts, our method can also accommodate zero-expressed gene counts and correct the confounding batch effects by introducing corresponding parameterisation into the ZINB mixture model. ZINBMM innovatively performs feature selection by imposing penalisation on the differences between cluster-specific and global mean values. Thus, the informative genes can be identified automatically in the clustering process, which cannot be achieved in most of the existing scRNA-seq data clustering studies, such as [6]. We demonstrate that ZINBMM brings significant advancements over competing state-of-the-art methods using both simulated and five real scRNA-seq datasets including embryonic stem cells, lung, uterus, liver, and mammary gland tissues from humans and mice.

## Results

### Overview of ZINBMM

**Fig. 1:**
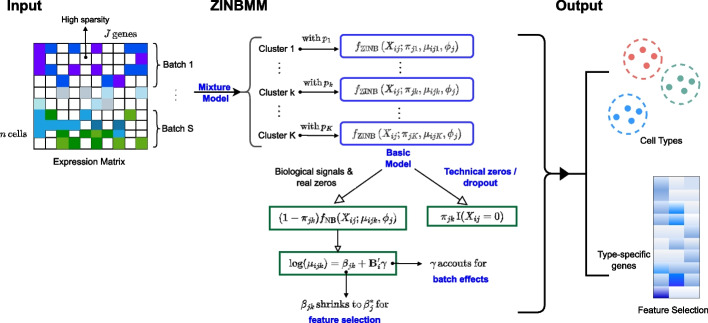
The schematic view of ZINBMM. With *n* cells and *J* genes, let $X_{ij}$ be the count expression of the $j^{\text {th}}$ $(j = 1,\dots ,J)$ gene from the $i^{\text {th}}$ $(i = 1, \dots , n)$ cell; $X_{ij}$ follows $f= \sum _{k=1}^{K}p_{k} \cdot f_\text {ZINB}\left( X_{ij};\pi _{jk},\mu _{ijk},\phi _j\right)$ with $f_{\text {ZINB}}(X_{ij}; \pi _{jk},\mu _{ijk},\phi _j)=\pi _{jk}\text {I}(X_{ij} = 0)+(1-\pi _{jk})f_{\text {NB}}(X_{ij};\mu _{ijk},\phi _j)$ and $\log (\mu _{ijk}) = \beta _{jk}+\varvec{B}'_i\varvec{\gamma }$. The final output of ZINBMM includes a cluster assignment for each cell and the selected cell type-specific genes

ZINBMM is a systemic model for the analysis of high-dimensional scRNA-seq data that have potential dropout events and batch heterogeneity. It can simultaneously identify cell types and cell type-specific genes. Herein, we briefly introduce the method. The technical details are provided in the “Methods” section, and a schematic view is presented in Fig. 1.

Consider *n* single cells and *J* genes, and denote $X_{ij}$ as the count expression of the $j^{\text {th}}$ $(j = 1,\dots ,J)$ gene from the $i^{\text {th}}$ $(i = 1, \dots , n)$ cell. We model $X_{ij}$ using a mixture of ZINB distributions, including a Bernoulli distribution for modelling zero-inflated dropout events and an NB distribution accounting for over-dispersed gene expression measurements with the count nature. The mixture model is developed to explicitly characterise cell type heterogeneity, where the probability mass function is defined as $\sum _{k=1}^{K}p_{k} \cdot f_\text {ZINB}(X_{ij};\pi _{jk},\mu _{ijk},\phi _j)$, with *K* being the number of mixture components (clusters) and $p_k$’s being the corresponding mixing proportions. Compared to the existing heuristic or geometric-based clustering methods [3, 13, 17], ZINBMM provides more intuitive interpretations from a statistical perspective by assigning cluster-specific probabilities to each cell. ZINBMM can also naturally model the heterogeneity of various cell types attributed to differential genes using cell type-specific mean parameters $\mu _{ijk}$’s, with the technical noises from dropouts eradicated using the zero inflation component.

To further control various technical batch effects, we propose a decomposition of $\mu _{ijk}$ and introduce a parameterisation $\varvec{\gamma }$ for joint batch effect removal. The inclusion of $\varvec{\gamma }$ enables batch effect correction within the clustering procedure, without the need for a prior correction step that is usually required in most alternative methods. Significantly advancing from the existing scRNA-seq data clustering analysis, the proposed method can also conduct cluster-specific gene identification, where the penalisation technique, with a solid ground in both theory and practice, is adopted. Specifically, the $L_1$ penalty is imposed to the difference between each cluster-specific value $\beta _{jk}$ and the global mean value $\beta _j^{*}$, which yields automatic gene selection through shrinking the estimate of $\beta _{jk}$ toward the global mean.

### Testing ZINBMM using simulated data

To evaluate the performance of ZINBMM in clustering and feature selection, we conduct extensive simulation studies. In brief, we consider three clusters and generate data from the ZINB mixture model using the baseline expression parameters obtained from the Zeisel dataset [23]. Dropout events are introduced using the zero-inflated parameters $\pi _{jk}$’s, which are generated from a Uniform distribution with different parameters, resulting in three different levels of dropout rates: about 15% (low), 45% (medium), and 75% (high). Two settings of batch parameters $\varvec{\gamma }$ and three settings of expression parameters $\varvec{\beta }$ are also considered, representing slight or large batch effects and low, medium, or high biological differences among clusters. More details are presented in the “Data simulation” section of the “Methods” section. Overall, the simulated experimental designs comprehensively cover a wide spectrum with various levels of dropouts, batch effects, and biological differences among clusters as well as both balanced and imbalanced sample distributions.

In addition to the proposed ZINBMM, we conduct analysis using nine competing clustering methods: CIDR [13], SC3 [3], Seurat [24], scDeepCluster [5], MNN-Graph [9, 25], MNN-Kmeans [9], RZiMM [6], snbClust [22], and sparseKmeans [26]. Among them, SC3 and Seurat are perhaps the most popular. CIDR, scDeepCluster, and RZiMM address the dropout problem. MNN-Graph, MNN-Kmeans, and RZiMM perform correction for batch effects. And RZiMM, snbClust, and sparseKmeans simultaneously conduct clustering and gene selection. Two other dropout-based gene selection methods, M3Drop and NBDrop [19], provide an additional comparison in terms of gene selection. We acknowledge the existence of other potential alternatives suitable for comparison. The above approaches are chosen owing to their popularity, competitive performance, and similar analysis frameworks. To evaluate clustering performance, we use adjusted Rand index (ARI). Moreover, Recall, Precision, and F1 are used to quantify gene selection performance (see Methods).

**Fig. 2:**
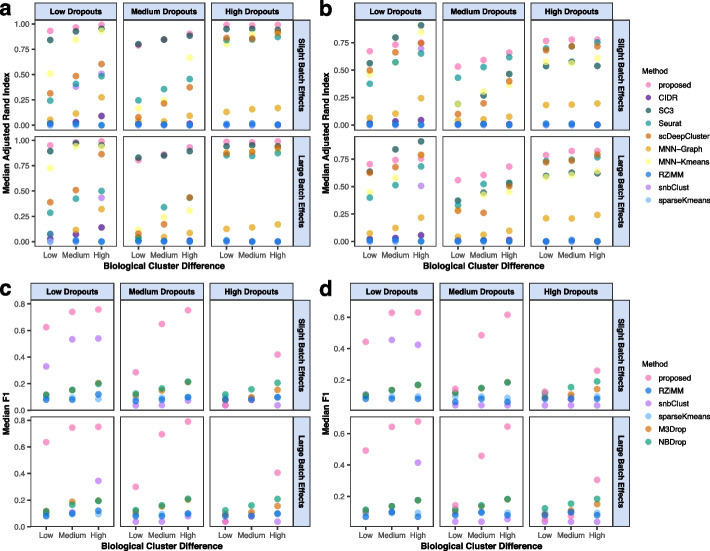
Simulation results with $K=3$. Plots of the median ARI under **a** a balanced sample distribution, **b** an imbalanced sample distribution, as well as median F1 under **c** a balanced sample distribution, and **d** an imbalanced sample distribution over 100 replicates. Different colours represent different methods.

A hundred replicates are simulated under each setting, and medians and median absolute deviations (MADs), as well as *P*-values computed from the Wilcoxon test for the proposed method and each alternative, are summarised. The median values of ARI and F1 are shown in Fig. 2, and the rest results are presented in Additional file 1: Fig. S1 and Additional file 2: Tables S1-S12. Here, the M3Drop and NBDrop methods’ ARI values are unavailable because they are primarily intended for gene selection and unable to identify cell clusters. Additionally, because the CIDR, SC3, Seurat, scDeepCluster, MNN-Graph, and MNN-Kmeans methods do not conduct gene selection, Recall, Precision, and F1 scores are not available for those methods.

As shown in Fig. 2a and b, ZINBMM exhibits favourable performance in terms of clustering accuracy across all simulation scenarios. Compared to the alternatives, it achieves a higher median ARI and the *P*-values computed from the Wilcoxon test are mostly $<0.05$ (Additional file 2: Tables S1–S12). Specifically, for the three levels of dropout rates, we follow the recent single-cell data analysis publications [27, 28] and consider the settings with dropout rates varying across clusters (see Methods). As pointed out in [29], the dropouts can also serve as informative signals for cell clustering, as the distributions of dropouts may vary across cell clusters. Thus, compared to the scenarios with a low and high dropout rate, all methods show a slightly worse clustering performance under the scenarios with a medium dropout rate. To get more intuitive insight, we consider three replicates simulated from the scenarios with three different levels of dropout rate but with the same biological cluster difference and batch effect. A special replicate with no dropouts is also considered. For these four replicates, the heatmaps of the 50 biologically important genes (with $\beta _{jk}$’s varying across clusters) and 20 none important genes and the boxplots of the mean expressions of the important genes and the proportions of zero observations in the three clusters are presented in Additional file 1: Fig. S2. It is observed in Fig. S2a that, although the biological cluster difference is fixed, the signals of biologically important genes vary across different dropout rates, which are covered by different ratios of zeros. In addition, in Fig. S2b, compared to the scenarios with a low and high dropout rate, the diversity of expression distributions across clusters is less significant under the scenario with a medium dropout rate, leading to the inferior clustering performance of the methods. Specifically, under the scenario with a low dropout rate, the diversity of expression distributions across clusters (*p*-value 0.00027) is mainly due to the biological cluster difference, which is not covered by the limited number of zeros (see the heatmap in Fig. S2a. Under the scenario with a medium or high dropout rate, biological information is somehow covered and confounded by dropout zeros (Fig. S2a). When the dropout rate is high, the differences across clusters are dominated by the distribution differences of zero observations across clusters, which are significant (*p* value 0.008) as shown in Fig. S2c, leading to significantly different mean expressions (*p* value 0.0071 in Fig. S2b). However, when the dropout rate is medium, both the differences of biological information (covered by dropout zeros) and dropout distribution (not strong enough to be distinctive with a *p* value of 0.65) across clusters are weak, leading to slightly worse clustering performance. Similar statements that dropouts can be informative have also been presented in [30] and [31].

In addition to the above analyses, we consider another setting, under which dropout rate is independent of cell types and thus dropouts are non-informative for cell clustering (see Methods), and observe that ARI values decrease as dropout rate increases (Additional file 2: Table S13). Performance of all methods generally decays as biological differences between clusters become smaller. However, the proposed method remains advantageous. Since both dropout distributions and biological information across cell types contribute to clustering, when dropout rate is high together with multiple levels of simulation randomness (e.g. top right panel of Fig. 2a), clustering performance does not significantly depend on biological cluster differences for some methods, such as the proposed and SC3 methods. The superiority of ZINBMM becomes more prominent under the imbalanced sample distribution (Fig. 2b), which is more common with practical data.

On the other hand, as seen in Fig. 2c and d, the advantage of the proposed method in gene selection is strongly evident. The F1 score, which is the weighted average of Precision and Recall, can offer a thorough assessment of gene selection. With higher levels of cluster differences, it is seen that the proposed method significantly improves gene selection performance. Even though the signals of important genes are severely hampered by the presence of high dropouts, where all methods have decreased F1 values, the proposed method is still far superior. As seen in Fig. S1a and b, NBDrop may have higher Recall values, primarily because it tends to choose more informative genes. In fact, the corresponding Precision values are only around 0.1 on average, compared to 0.75 of the proposed method.

We also perform simulations with various values of *K* (Additional file 1: Fig. S3 and Additional file 2: Tables S14-S18). It is observed that as the number of clusters increases, most methods perform worse as the underlying heterogeneity is more complex. When $K=4, 5, 8,$ and 10, all the informative genes are simulated to express both up-regulation $(\delta = 1)$ and down-regulation $(\delta = -1)$ across clusters (in comparisons with mostly up-regulated genes when $K=3$), which is much more friendly to the pairwise comparison scheme of RZiMM. As a result, compared to $K=3$, performance of RZiMM increases when the dropout rate is low, with the biological cluster effects not covered by the dropout zeros, but is similarly unsatisfactory when the dropout rate increases. In most cases, the proposed method is still superior in both clustering and gene selection accuracy. In Table S18 (Additional file 2) with a fixed sample size $n=600$, when *K* is larger (6 and 8) with more unknown parameters and the sample size of each cluster is limited, clustering performance of the proposed method is slightly worse than SC3. However, such performance can be easily enhanced by a larger sample size. The current simulation studies show that 150 samples per cluster can already provide satisfactory results, which can be easily satisfied with practical data.

Overall, the proposed method can achieve superior performance in both clustering and gene selection, and the significance of improvement is supported by statistical testing. SC3 achieves the second best clustering accuracy. Under some settings with low levels of dropouts, SC3 behaves slightly better than the proposed, perhaps because of the combined clustering strategy. However, it gets less effective when there are higher levels of dropouts. RZiMM achieves the second best gene selection accuracy, since it also accommodates dropouts, batch effects, and cell heterogeneity. The superiority of the proposed method over CIDR, Seurat, snbClust, and sparseKmeans directly supports the zero-inflation-based strategy for accommodating for dropout events. Improved performance over RZiMM and scDeepCluster suggests the effectiveness of the proposed penalisation scheme. Moreover, the proposed method performs much better than two MNN-based methods, partially suggesting the potential loss of information and bias introduction through multi-staged processing. Without an effective accommodation of cell heterogeneity, the two dropout-based gene selection methods M3Drop and NBDrop have unsatisfactory selection performance.

We further evaluate stability of the proposed method and examine whether it can maintain its superiority when the data generation model is misspecified. In particular, we consider three types of models: (a) the negative binomial mixture model considered in snbClust [22] without dropouts and batch effects, (b) the mixture zero-inflated Poisson model considered in RZiMM [6] in which a randomly selected cell type is designed to have a higher mean expression than the other types, and (c) the mixture zero-inflated Poisson model adopted in CIDR [13] in which the levels of dropouts are inversely proportional to the expression levels following a decreasing logistic function. Summary results are presented in Table 1 (results for MNN-Graph and MNN-Kmeans are not available under the models considered in snbClust and CIDR without batch effects). It is not surprising that the proposed method has a slightly worse performance compared to the method that matches the data generation model. For example, under the NB mixture model without dropouts and batch effects (snbClust), the median ARI values of snbClust and the proposed method are 0.627 and 0.564, with *P*-value computed from the Wilcoxon test being 0.946. However, the proposed method still outperforms the other competing ones. The majority of the alternatives behave poorly under the RZiMM setting, probably due to the presence of multiple batch effects. On the other hand, under the CIDR setting, the inverse relationship between dropouts and expression levels enhances the differences among differentially expressed genes, leading to satisfactory clustering accuracy for most of the methods.

**Table 1:** Simulation results under different model assumptions. In each cell, median (MAD) is based on 100 replicates, and *P*-value is computed from the Wilcoxon test for the proposed method and each competing method

Method	ARI		Recall		Precision		F1	
	Median (MAD)	*P*-value	Median (MAD)	*P*-value	Median (MAD)	*P*-value	Median (MAD)	*P*-value
snbClust								
Proposed	0.564 (0.123)	-	0.940 (0.040)	-	0.430 (0.026)	-	0.575 (0.027)	-
CIDR	0.128 (0.065)	0.000	- -	-	- -	-	- -	-
SC3	0.288 (0.102)	0.000	- -	-	- -	-	- -	-
Seurat	0.462 (0.050)	0.000	- -	-	- -	-	- -	-
scDeepCluster	0.472 (0.056)	0.002	- -	-	- -	-	- -	-
RZiMM	0.464 (0.404)	0.099	0.890 (0.070)	0.000	0.250 (0.220)	0.012	0.547 (0.093)	0.027
snbClust	0.627 (0.268)	0.946	0.980 (0.020)	0.915	0.268 (0.137)	0.000	0.391 (0.175)	0.000
sparseKmeans	0.001 (0.002)	0.000	1.000 (0.000)	1.000	0.050 (0.000)	0.000	0.095 (0.000)	0.000
M3Drop	- -	-	0.290 (0.030)	0.000	0.100 (0.012)	0.000	0.148 (0.017)	0.000
NBDrop	- -	-	0.280 (0.040)	0.000	0.104 (0.014)	0.000	0.152 (0.020)	0.000
RZiMM								
Proposed	0.897 (0.103)	-	1.000 (0.000)	-	0.573 (0.080)	-	0.712 (0.060)	-
CIDR	0.008 (0.007)	0.000	- -	-	- -	-	- -	-
SC3	0.000 (0.001)	0.000	- -	-	- -	-	- -	-
Seurat	0.034 (0.029)	0.000	- -	-	- -	-	- -	-
scDeepCluster	0.014(0.014)	0.000	- -	-	- -	-	- -	-
MNN-Graph	0.001 (0.001)	0.000	- -	-	- -	-	- -	-
MNN-Kmeans	0.002 (0.001)	0.000	- -	-	- -	-	- -	-
RZiMM	1.000 (0.000)	0.999	1.000 (0.000)	0.041	0.883 (0.000)	1.000	0.909 (0.000)	1.000
snbClust	− 0.001 (0.001)	0.000	0.165 (0.165)	0.000	0.055 (0.005)	0.000	0.097 (0.010)	0.000
sparseKmeans	0.069 (0.012)	0.000	0.405 (0.385)	0.000	0.631 (0.369)	0.729	0.179 (0.031)	0.000
M3Drop	- -	-	0.695 (0.025)	0.000	0.188 (0.007)	0.000	0.297 (0.012)	0.000
NBDrop	- -	-	1.000 (0.000)	1.000	0.100 (0.000)	0.000	0.182 (0.000)	0.000
CIDR								
Proposed	0.965 (0.035)	-	0.990 (0.010)	-	0.718 (0.036)	-	0.820 (0.017)	-
CIDR	0.999 (0.001)	0.994	- -	-	- -	-	- -	-
SC3	1.000 (0.000)	1.000	- -	-	- -	-	- -	-
Seurat	1.000 (0.000)	1.000	- -	-	- -	-	- -	-
scDeepCluster	0.570 (0.138)	0.000	- -	-	- -	-	- -	-
RZiMM	0.957 (0.043)	0.015	0.880 (0.040)	0.000	0.587 (0.027)	0.000	0.704 (0.032)	0.000
snbClust	0.954 (0.046)	0.028	0.955 (0.015)	0.000	0.977 (0.010)	1.000	0.964 (0.006)	1.000
sparseKmeans	0.913 (0.041)	0.000	0.915 (0.085)	0.001	0.192 (0.092)	0.000	0.193 (0.000)	0.000
M3Drop	- -	-	0.810 (0.020)	0.000	0.147 (0.004)	0.000	0.248 (0.007)	0.000
NBDrop	- -	-	0.990 (0.010)	0.139	0.109 (0.001)	0.000	0.196 (0.001)	0.000

To get more intuitive insight into the impact of batch effects and better appreciate the operating characteristics of the proposed method, we additionally conduct batch correction for SC3 and Seurat using the Harmony approach [32], under the scenarios where batch effects are relatively large and easier to correct for. It is noted that among the eleven competing methods, MNN-Graph, MNN-Kmeans, and RZiMM are already able to accommodate the batch effects. In addition, since the batch correction step (such as Harmony) usually involves data transformation and/or dimension reduction and makes corrected data continuous, it is not feasible to introduce a batch correction step for the sparseKmeans, M3Drop, and NBDrop methods, which conduct gene selection, and the CIDR, scDeepCluster, and snbClust methods, which rely on raw count or CPM data structure. The corresponding clustering results are presented in Additional file 2: Table S19. It is observed that under some scenarios with relatively low dropout rates, the median ARI value of Seurat is improved by batch correction but still much smaller than that of the proposed method (for example, increased from 0.424 to 0.796 compared to 0.980 of the proposed method under the scenario with a low dropout rate and a balanced sample distribution). On the other hand, when dropout rate is high, the ARI values of both SC3 and Seurat decrease, which may be attributed to bias caused by the PCA embedding of Harmony. In fact, as demonstrated in [32], Harmony tends to have poor performance with lowly expressed data. This analysis supports the validity of the proposed strategy for accommodating batch effects, dropouts, and high dimensionality simultaneously.

Finally, we examine computational efficiency of the proposed method under the above simulation settings with various numbers of cells, genes, and clusters. All analysis is conducted using a computer with an Intel Core i5 processor and 16 GB RAM, and the average computer time with fixed tuning parameters is provided in Table S20 (Additional file 2). As M3Drop and NBDrop only do gene selection but do not take clustering analysis into account, they are found to be much faster than the other ten methods. In general, the proposed method requires heavier computing than some graph-based (e.g. MNN-graph and Seurat) and geometric-based (e.g. MNN-Kmeans and CIDR) clustering methods, where dimension reduction is applied and low-dimensional factors are analysed in clustering analysis. However, when compared to the more direct competitors, scDeepCluster, RZiMM, and snbClust (which are similarly model-based and work on raw counts), and the most popular scRNA-seq clustering method SC3, the proposed method is observed to be competitively efficient. We additionally apply the proposed method to a simulated dataset with 100,000 cells, and the proposed analysis takes about 9.8 h, indicating its computational feasibility even for large-scale datasets.

### Applying ZINBMM to real scRNA-seq datasets

We consider five publicly available scRNA-seq datasets, on mouse embryonic stem cells (mESCs) [33], human lung adenocarcinoma (LUAD) cell lines [34], uterus, liver, and mammary gland cells from the Mouse Cell Atlas (MCA) [35]. In all of these five datasets, each cell has been annotated by cell and lineage markers, and the annotations of cells and cell type memberships have been treated as gold standards to facilitate objective clustering comparisons. Summary information, including the numbers of cells and genes, proportion of zeros, number of cell types, and batch information, is provided in Table 2.

**Fig. 3:**
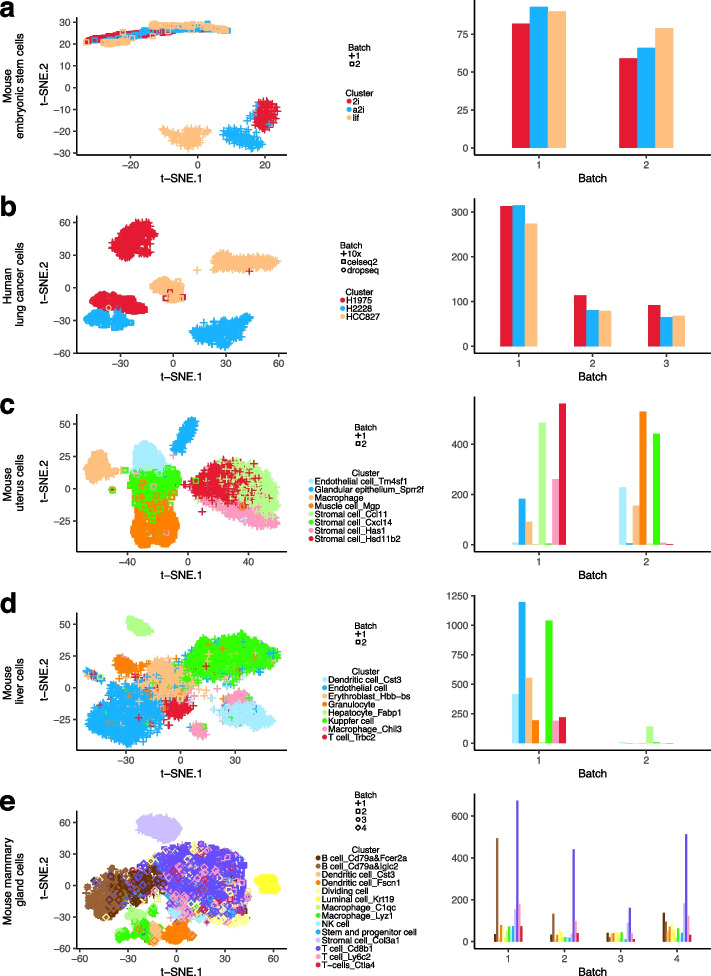
Visualization of the five real datasets. Two-dimensional t-SNE projection of cells (coloured and shaped by different types and batches) and compositional bar plots of cell types among different batches for **a** mouse embryonic stem cells, **b** human lung cancer cells, **c** mouse uterus cells, **d** mouse liver cells, and **e** mouse mammary gland cells

To obtain deeper insights into batch characteristics, we further provide a visualized illustration of these datasets in Fig. 3. Specifically, the raw count data is projected using the t-SNE method [36] and then mapped onto a two-dimensional space. The related t-SNE plots are shown in the left panel of Fig. 3, with different cell types and batches illustrated using different colours and shapes. In addition, the proportions of cell types in different batches are presented in the right panel of Fig. 3. In the mouse embryonic stem cell dataset, the differences between cell types are dominated by batch effects. Specifically, the cells from batch 1 (plus-shaped) gather at the bottom right, whereas cells from batch 2 (square-shaped) gather at the top left. In addition, Fig. 3b shows that the human lung cancer cells are well separated with regard to both protocol differences and cell types. The mouse uterus dataset represents a more complex situation wherein there is some confounding between batch and biology, as shown in Fig. 3c. The mouse liver dataset represents a special case wherein batch effect contributes to cell-type differences. Specifically, the second batch almost only contains cells from Hepatocyte_Fabp1, which separately gather at the top left as illustrated in Fig. 3d. The mouse mammary gland dataset (Fig. 3e) is a large-scale dataset with larger numbers of clusters and batches, where the distributions of both clusters and batches are highly imbalanced. Overall, as illustrated in Table 2 and Fig. 3, the five datasets comprehensively cover a wide spectrum with different levels of dropout rates as well as diverse batch effect types.

For each dataset, in principle, the proposed method can be directly applied. However, considering that only a small number of genes are potentially associated with the formation of cell types, we conduct a pre-screening by selecting the top 1,000 highly variable genes (HVGs) with the largest standard deviations for downstream analysis. As stated in [37], this screening step can amplify underlying information in the retained genes while significantly reducing computational cost. The choice of 1,000 HVGs has been widely adopted in scRNA-seq clustering [11, 38, 39]. More details regarding the collection and processing of data are provided in the “Real scRNA-seq datasets” section of the “Methods” section.

**Table 2:** Summary information of the five real datasets

Dataset	No. of cells	No. of genes	Proportion of zeros	No. of cell types	Batch information
Mouse embryonic stem cells	469	38,658	71.22%	3	Factorial design with balanced distribution
Human lung cancer cells	1402	12,997	41.16%	3	Factorial design from different protocols
Mouse uterus cells	2965	8994	91.47%	8	Confounding factors between batch and biology
Mouse liver cells	3971	8868	93.60%	8	Slight batch influence
Mouse mammary gland cells	5154	8254	93.72%	14	Multiple batches

### Examination of the number of clusters

We consider the following candidate sequence: $K=\{2,3,\cdots ,19,20\}$. For the proposed method, the modified Bayesian Information Criterion (BIC) is adopted. For the nine alternatives, we use the criteria recommended by the corresponding published studies: Calinsk-Harabasz index for CIDR [13], random matrix theory (RMT) for SC3 [3], resolution parameter-based strategy for Seurat [24], elbow-method strategy for scDeepCluster [5], modularity maximisation for MNN-Graph [25], modified BIC for RZiMM [6], BIC for snbClust [22], and Gap statistic for MNN-Kmeans and sparseKmeans [26].

The results are presented in Table 3. For the mouse embryonic stem cell and human lung cancer cell datasets, which have relatively small numbers of cell types, the proposed method can correctly identify the true numbers of cell types. For the mouse uterus, liver, and mammary gland cell datasets, the proposed method identifies five, four, and five clusters, respectively, and some cell types with a certain similarity are clustered into large cell types. For example, for the mouse uterus cell dataset, three types Cxcl14, Has1 and Hsd11b2 from Stromal are grouped into two types. In addition, for the mammary gland cell dataset, two B cell subtypes and three T cell subtypes are clustered into two groups separately. As shown in Table 3, few other methods can identify the true numbers of cell types. Specifically, SC3, Seurat, and scDeepCluster tend to choose larger numbers of clusters, while the optimal numbers chosen by Gap statistics for the two Kmeans-based methods are small. For making a fair comparison, we set *K* as the true number of cell types for all methods in the following analysis, which is a common practice in recent clustering analysis of scRNA-seq data [5, 22].

**Table 3:** Optimal numbers of cell types identified using different methods for the five real datasets

Method	True	Proposed	CIDR	SC3	Seurat	scDeepCluster	MNN-Graph	MNN-Kmeans	RZiMM	snbClust	sparseKmeans
Dataset											
Mouse embryonic stem cells	3	3	5	5	6	7	4	3	2	2	5
Human lung cancer cells	3	3	6	12	14	12	2	2	5	4	2
Mouse uterus cells	8	5	12	11	10	9	7	2	8	7	2
Mouse liver cells	8	4	12	12	12	11	7	2	7	8	2
Mouse mammary gland cells	14	5	8	16	15	17	16	2	4	6	2

### ZINBMM leads to biologically sensible clusters

**Fig. 4:**
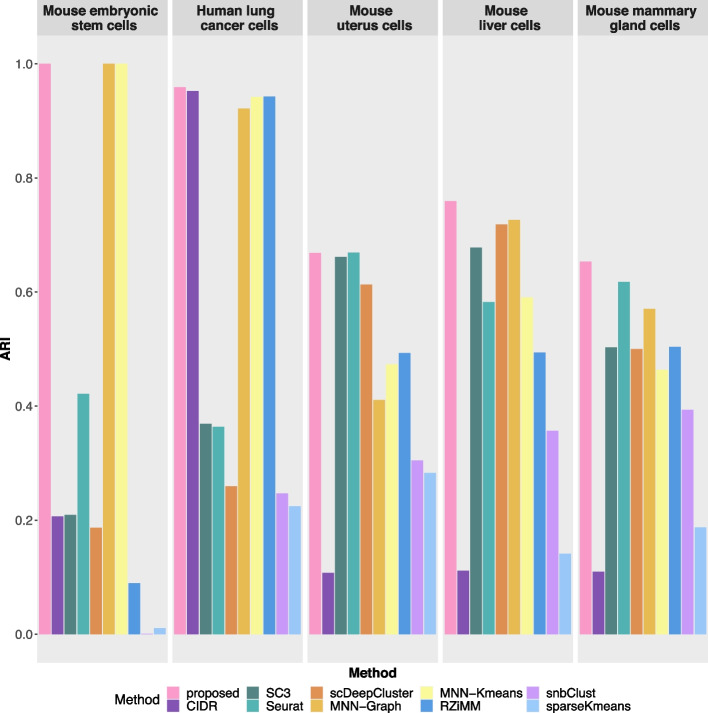
Clustering performances measured using ARI for the five real datasets

**Fig. 5:**
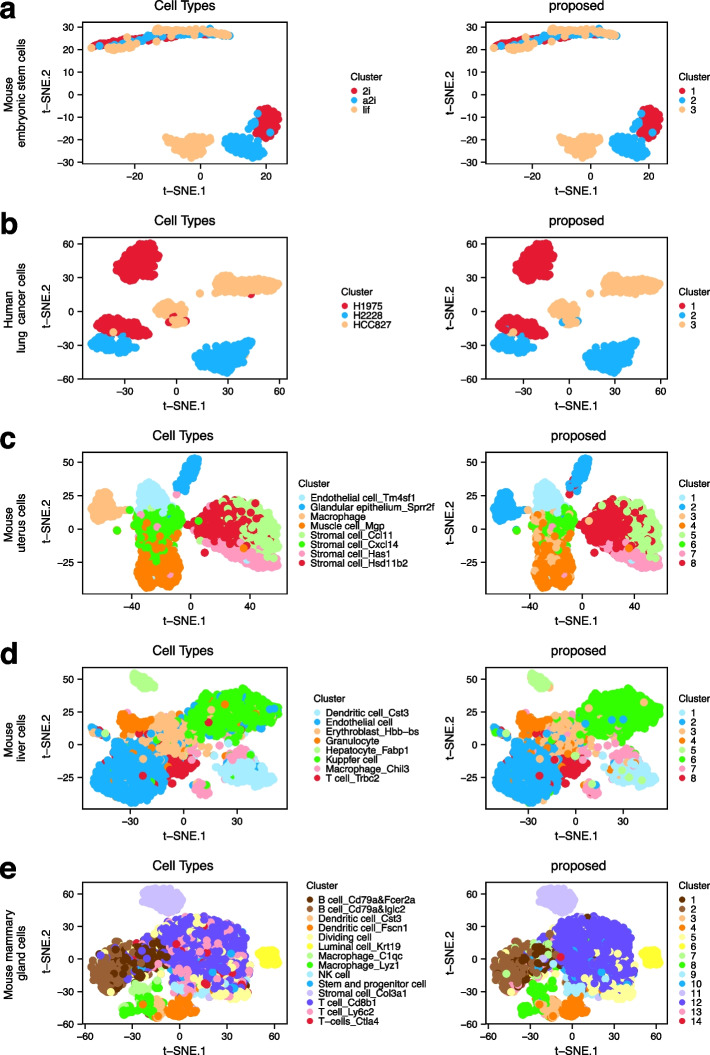
Clustering performance of the proposed method for the five real datasets. Two-dimensional t-SNE projection of cells (coloured based on the annotated cell types and clustering results identified using the proposed method) for **a** mouse embryonic stem cells, **b** human lung cancer cells, **c** mouse uterus cells, **d** mouse liver cells, and **e** mouse mammary gland cells

We conduct analysis using the proposed method as well as the nine alternatives and present the ARI values in Fig. 4. The proposed method consistently outperforms the other nine clustering methods. We also present the t-SNE projection plots wherein each cell is coloured by its annotated cell type (Fig. 5, left) and the cluster label identified using the proposed method (Fig. 5, right), respectively. For each dataset, a high similarity between these two plots is observed. Similar t-SNE projection plots for the nine alternatives are presented in Additional file 1: Figs. S4–S8. The alternatives cannot effectively identify the true patterns of cell types occasionally. First of all, the presence of a strong batch effect in the mouse embryonic stem cell dataset poses a problem for the methods without accommodation of batch effects. However, the design in which each batch includes cells from each biological condition favours an effective batch effect correction, making the proposed and two MNN-based batch correction methods able to identify cell types perfectly. Second, in the human lung cell dataset, the low level of dropouts and high level of variations with regards to both batches and cell types lead to a satisfactory clustering accuracy for the proposed method, as well as for the CIDR, RZiMM, MNN-Graph, and MNN-Kmeans methods. Third, under a more complex setting wherein a high level of dropouts and confounding factors (mouse uterus cells) are present, a special case with small batch effects (mouse liver cells), and a larger dataset with higher numbers of clusters and batches (mouse mammary gland cells), the proposed method is observed to have much greater advantages in terms of clustering accuracy. Overall, the methods that cannot handle dropouts, such as snbClust and sparseKmeans, always have inferior performance. In addition, without an adjustment for batch effects, the clustering patterns identified by CIDR, SC3, and Seurat are likely to be dominated by batch effects rather than heterogeneity. The proposed method also behaves much better than RZiMM and scDeepCluster owing to the effective penalised feature selection.

### ZINBMM selects genes associated with cell types

Besides cell types, the proposed method also identifies 33, 24, 51, 55, and 80 important cell type-associated genes for the mouse embryonic stem cell, human lung cancer cell, mouse uterus cell, mouse liver cell, and mouse mammary gland datasets, respectively. We also conduct gene selection using RZiMM, snbClust, sparseKmeans, M3Drop, and NBDrop. The summary results are shown in Additional file 2: Tables S21–S25, where the numbers of genes identified with these methods and their overlaps are presented. Because RZiMM provides gene importance score instead of gene selection results, we focus on the top 50 genes with the largest scores. Tables S21–S25 provide information on the differences and similarities between the findings. It is observed that different methods identify different sets of genes with moderate overlapping.

**Fig. 6:**
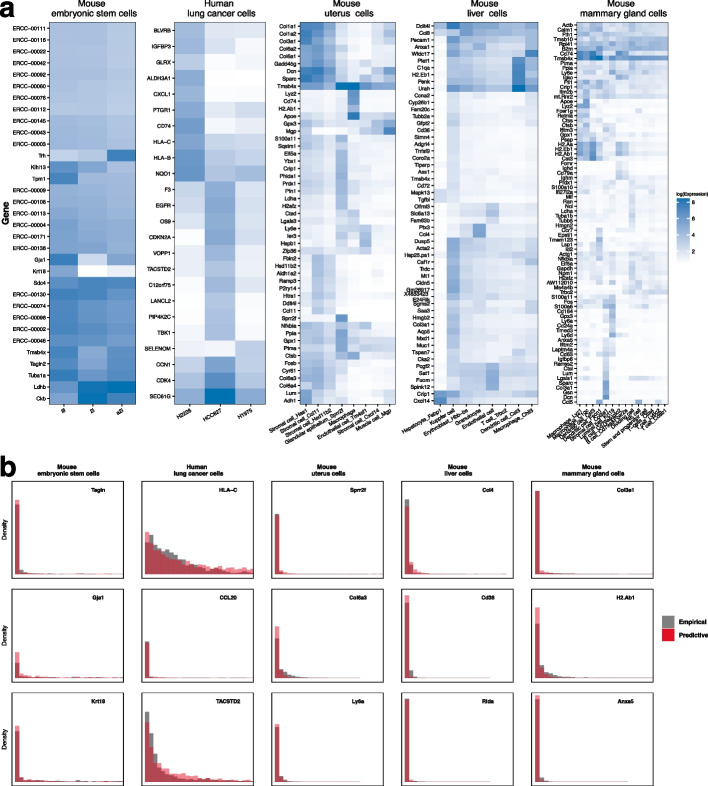
Gene selection performance of the proposed method for the five real datasets. **a** Log-transformed expressions of the identified genes among different true cell types. **b** Predictive and empirical data distributions of representative genes

To obtain insights into the selected genes using the proposed method, we present an expression heatmap based on the ground true cell types in Fig. 6a. The corresponding heatmap plots of the selected genes using the alternative methods are shown in Additional file 1: Figs. S9–S13. The methods with extremely large numbers of selected genes are not presented. Compared with the alternatives, evident differences are noted in the expression levels among different cell types using the proposed method, which supports significance of the selected genes. Consider gene Krt18 in the mouse embryonic stem cell dataset as an example. This gene is highly expressed in the cell type “lif”, whereas its expression is extremely low in the other two cell types. Additionally, for the human lung cell dataset, a group of low-expressed genes is detected in the cell type “H1975”, and notably different sets of highly-expressed genes are detected in the cell types “H2228” and “HCC827”. We note that for the mouse uterus dataset with a relatively lower ARI value (0.668), the proposed method also has satisfactory gene selection performance as shown in Fig. 6a. In fact, true cluster labels are identified for almost 75% of the cells. ZINBMM is able to effectively capture biological information from these appropriately assigned cells while remaining resistant to the misclassified cells. In addition to heatmap, for each dataset, we consider three important genes as examples and show predictive data distribution based on the proposed ZINBMM and estimated parameters as well as the empirical data distribution in Fig. 6b. The predictive data distributions are observed to fit the empirical data very well, regardless of whether the genes have a high (e.g. Tagln) or a low level of dropouts (e.g. HLA-C). This indicates that ZINBMM can effectively model technical zeros and biological zeros as well as biological effects while simultaneously accommodating cell heterogeneity and batch effects.

Literature search suggests that many of the identified genes have strong biological implications for cell types. For example, for the mouse embryonic stem cell dataset, the high expression of gene Krt18 has been found to contribute to the formation of a minor formative-state pluripotent population [40]. Gene GJA1 has been implicated as one of the markers in [41] for quiescent nonspecific conversion pioneer factors (qNSCs) to induce pluripotency in mouse embryonic stem cells. Gene Trh is a unique endoderm marker that transiently marks the entire definitive endoderm population and is not expressed in the extraembryonic endoderm. In the human lung cell dataset, gene CD74 has been shown to play an important role in eliciting immune response in lung adenocarcinoma [42]. CDKN2A has been identified as a tumour suppressor associated with the detection of regulatory gene hubs [42]. Gene EGFR has been commonly used as an important therapeutic target for non-small-cell lung carcinoma (NSCLC) [43]. Among the genes detected in the mouse uterus cell dataset, Col1a1 encodes collagen and laminin, the high expressions of which have been reported in adventitial stromal cells [44, 45]. Gene CD74 has been shown to contribute to macrophage separation [46]. Preferential expression of gene Sprr2f has been found conducive to epithelial cluster formation in uterus [47]. For the mouse liver cell dataset, gene C1qa is a commonly used biomarker, and the high expression of C1qa has been identified as a tumour-specific signature [48]. Gene CD36 has been suggested as a regulator of Kupffer cell metabolism [49]. Highly upregulated Col3a1 has been identified to be particularly important for spatial heterogeneity across liver tissues [50]. For the mouse mammary gland dataset, gene Lyz1 has been implicated as a marker gene for myeloid cells in mammary glands [51]. Mif has been identified as a lineage-specific gene with a strong correlation with stem pseudotime in mammary epithelium cells [52]. Expression of gene Apoe has been found to help discriminate tumour-associated macrophages in breast tumours [53].

**Fig. 7:**
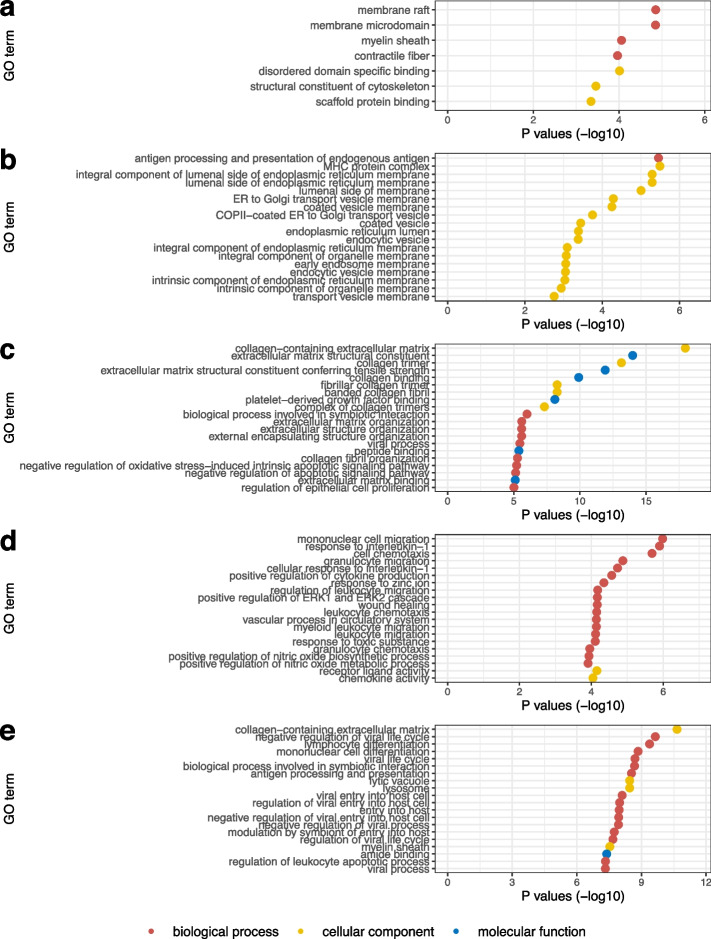
GO enrichment analysis with reference *P* values of the selected genes for the five datasets: **a** Mouse embryonic stem cells, **b** human lung cancer cells, **c** mouse uterus cells, **d** mouse liver cells, and **e** mouse mammary gland cells

To provide an additional indicator for the quality of gene selection, we further analyse biological relevance of the selected genes by conducting gene ontology (GO) enrichment analysis. The analysis is conducted to evaluate molecular functions, cellular components, and biological processes of the selected genes. The results are shown in Fig. 7. Significantly enriched categories are observed, with distinct categories across the five datasets. Specifically, seven significantly enriched terms are observed from Fig. 7a for mouse embryonic stem cells, including membrane raft, myelin sheath, disordered domain specific binding and structural constituent of cytoskeleton. One biological process term and multiple cellular component terms are significantly enriched for human lung cancer cells, as observed in Fig. 7b, including coated vesicle membrane, endoplasmic reticulum lumen and intrinsic component of the endoplasmic reticulum membrane. A total of 60 significantly enriched GO terms are observed for mouse uterus cells, and we show the top 20 in Fig. 7c. The results suggest that these genes are significantly contributed to extracellular organisation and molecular binding. The enriched terms of the detected genes in liver cells are associated with biological migration and cellular activity, as shown in Fig. 7d. The top 20 significant GO terms for mouse mammary gland cells are presented in Fig. 7e, where the associations with different processes and functionalities are observed. Overall, the significance of the associated GO categories supports the validity of the proposed gene selection procedure.

## Discussion

Advances in single-cell technologies have enabled the measurement of gene expressions in massive individual cells, providing opportunities for a better understanding cellular heterogeneity. However, scRNA-seq experiments suffer from severe batch effects and dropout events, making read count data noisy and sparse. Furthermore, these experiments are characterised as high dimensional, because they typically measure the expressions of tens of thousands of genes. Even after pre-processing, the remaining genes may still contain redundant information. These factors call for the development of effective statistical models that can account for high-dimensional scRNA-seq data clustering with batch effects and zero inflation.

We have developed ZINBMM, incorporating a penalisation technique that simultaneously achieves clustering and gene selection. ZINBMM directly models raw count data without transformation. The mixture model can naturally describe batch effects and dropout events, facilitating a biologically interpretable clustering analysis. Furthermore, with penalisation on the differences between global and cluster-specific means, ZINBMM can conduct cluster-discriminatory gene selection, improving clustering accuracy and biological understanding. Comprehensive evaluations and comparisons with state-of-the-art methods on both simulation and real data applications have been conducted. ZINBMM has demonstrated stable and superior results over the alternatives.

We have developed a two-layer mixture model, where the first and second layers have been proposed to accommodate cell heterogeneity and zero-inflated values. The identifiability of mixture distributions is unfortunately still a wide-open problem [54]. In published studies, the identifiability of the NB mixture model and the zero-inflated model has been well established. Moreover, the two-layer mixture model has been common in recent studies [55], where a two-layer EM algorithm has been adopted for optimization. For one simulated replicate, we have conducted the proposed analysis multiple times and obtained the same estimator, which may suggest the identifiability of the proposed method. In this study, we have mostly focused on methodological development and implementation. Theoretical studies on identifiability may be beyond the scope of this study. We have introduced the parameter vector $\varvec{\gamma }$, which accommodates the linear batch effects and may have been overly simplified when compared to what is practically observed. We have adopted this strategy to enhance computation efficiency, and it has been demonstrated to exhibit satisfactory performance in both simulation and data analysis with various types of batch effect patterns. This strategy may be of interest in extending the proposed framework to accommodate nonlinear batch effects using non-parametric techniques. This study has focused on the heterogeneity of cell types. The proposed method has the potential to be extended to account for both individual- and cell-level heterogeneity by adding more parameters.

We have conducted analysis with 1000 HVGs to improve performance and reduce computational cost. Additionally, we have also examined the results with 2000 HVGs. The comparison is provided in Additional file 2: Table S26. It is observed that both the clustering and gene selection results do not significantly depend on the number of input genes. More rigorous investigation on the impact of the number of input genes will be pursued in future work. We have adopted the modified BIC to select the optimal number of cell clusters, and it has been the most popular in published model-based clustering analysis studies [6, 22]. For the datasets with a larger number of cell types, as the modified BIC tends to select models with a relatively smaller number of parameters, a smaller number of cell types is usually identified and some cell types are clustered into a larger one. If there are reasons to believe that a sample is heterogeneous or if one is interested in uncovering new subtypes, it is convenient for the proposed method to further cluster cells based on the existing results. Additional investigation on the optimal number of clusters is deferred to further study. In data analysis, we have made findings different from those of the alternatives. Literature search and GO enrichment analysis have shown their important biological implications. A more definitive confirmation from functional validations will be needed.

## Conclusion

In this work, we have developed a novel statistical method ZINBMM to conduct simultaneous clustering and cluster-specific gene identification for scRNA-seq data, specifically to address the challenges of batch effects and dropout events. Experiment results on both simulation and five real datasets have demonstrated that the proposed method significantly improves clustering and gene selection performance compared to the alternatives. Motivated by the importance of clustering analysis for high-dimensional scRNA-seq data, ZINBMM is a valuable tool for elucidating cellular heterogeneity and providing biological insights into the underlying mechanism.

## Methods

### Statistical model

Let $X_{ij}$ be the expression of the $j^{\text {th}}$ gene from the $i^{\text {th}}$ cell, for $i = 1, \dots , n, j = 1,\dots ,J$. For the $i^{\text {th}}$ cell, to account for heterogeneity and dropouts in the single-cell expression count data, we assume that the expression $X_{ij}$ follows a mixture zero-inflated negative binomial distribution. The probability mass function is as follows:

1 $$\begin{aligned} f(X_{ij};\varvec{\theta })= & {} \nonumber \sum _{k=1}^{K}p_{k} \cdot f_\text {ZINB}(X_{ij};\pi _{jk},\mu _{ijk},\phi _j),\\= & {} \sum _{k=1}^{K}p_{k} \cdot \left[ \pi _{jk}\text {I}(X_{ij} = 0)+(1-\pi _{jk})f_{\text {NB}}(X_{ij};\mu _{ijk},\phi _j)\right] , \end{aligned}$$

with $f_{\text {NB}}(X_{ij};\mu _{ijk},\phi _j) = \frac{\Gamma (X_{ij}+\phi _j)}{X_{ij}!\,\Gamma (\phi _j)}\left( \frac{\mu _{ijk}}{\mu _{ijk}+\phi _j}\right) ^{X_{ij}}\left( \frac{\phi _j}{\mu _{ijk}+\phi _j}\right) ^{\phi _j}$ and $\text {I}(\cdot )$ being the indicator function. Here, *K* is the number of cell types (mixture components), $\varvec{p} =(p_1,\dots ,p_K)'$ is the vector of mixing proportions satisfying $p_k\ge 0$ and $\sum _{k=1}^{K}p_k = 1$, $\pi _{jk}$ is the probability that the $j^{\text {th}}$ gene in the $k^{\text {th}}$ cluster expresses zero caused by dropouts, and $\mu _{ijk}$ and $\phi _j$ are the mean and dispersion parameters of the negative binomial distribution. To adjust for batch effects, we further parameterize the mean value $\mu _{ijk}$ as:

$$\begin{aligned} \log (\mu _{ijk}) = \beta _{jk}+\varvec{B}'_i\varvec{\gamma }, \end{aligned}$$

where $\beta _{jk}$ is the mean expression of the $j^{\text {th}}$ gene in the $k^{\text {th}}$ cluster on the log scale after controlling for batch effects, $\varvec{B}_i = (B_{i1},\dots ,B_{iS})'$ is the indicator vector corresponding to the batches with $B_{is} = 1$ indicating that the $i^{\text {th}}$ cell belongs to the $s^{\text {th}}$ batch and *S* being the number of batches known in advance, and $\varvec{\gamma }=(\gamma _1,\dots ,\gamma _S)'$ is the parameter vector of batch effects. Overall, the vector of unknown parameters $\varvec{\theta }$ includes all $p_k$’s, $\pi _{jk}$’s, $\beta _{jk}$’s, and $r_s$’s.

For regularised estimation and gene selection, we propose the following penalised objective function:

2 $$\begin{aligned} l_p(\varvec{\theta })= & {} \sum _{i=1}^{n} \log \left[ \sum _{k=1}^{K} p_{k} \cdot \prod _{j=1}^{J} f_{\text {ZINB}}\left( X_{ij}; \pi _{jk},\exp \left( \beta _{jk}+\varvec{B}'_i\varvec{\gamma }\right) ,\phi _j\right) \right] -\lambda \sum _{j=1}^{J}\sum _{k=1}^{K}\left| \beta _{jk}-\beta _j^{*} \right| \nonumber \\\triangleq\;& {} l(\varvec{\theta })-\lambda \sum _{j=1}^{J}\sum _{k=1}^{K}\left| \beta _{jk}-\beta _j^{*} \right| , \end{aligned}$$

where $\beta _j^{*}$ is the pre-estimated global expression measurement of the $j^{\text {th}}$ gene assuming no cluster effects after controlling for batch effects, and $\lambda$ is a tuning parameter. In (2), the first term is the log-likelihood function, whereas the second term is proposed to identify cell type-specific genes, wherein we impose regularisation on the difference between $\beta _{jk}$ and $\beta _j^{*}$ to promote $\beta _{jk}$ to shrink to the global value $\beta _j^{*}$. By maximising (2), gene *j* that has all $\beta _{j1}, \dots , \beta _{jK}$ equal to $\beta _j^{*}$ will be identified as an unimportant gene. The important genes, on the other hand, are those with at least one $\beta _{jk}$ different from $\beta _j^{*}$.

### Optimisation

We adopt an expectation-maximisation (EM) algorithm for the maximisation of (2). First, an unobserved indicator $z_{ik}$ is introduced for the $i^{\text {th}}$ cell, where $z_{ik} = 1$ if the $i^{\text {th}}$ cell belongs to the $k^{\text {th}}$ cell type and $z_{ik} = 0$ if otherwise. Then, based on (2), the complete-data objective function is:

3 $$\begin{aligned} l_c(\varvec{\theta })= & {} \sum _{k=1}^{K} \sum _{i=1}^{n} z_{i k}\left[ \log p_{k}+\sum _{j=1}^{J} \log f_{\text {ZINB}}\left( X_{ij}; \pi _{jk},\exp \left( \beta _{jk}+\varvec{B}'_i\varvec{\gamma }\right) ,\phi _j\right) \right] \nonumber \\{} & {} -\lambda \sum _{j=1}^{J}\sum _{k=1}^{K}\left| \beta _{jk}-\beta _j^{*} \right| . \end{aligned}$$

With (3) and a fixed tuning parameter, the optimisation proceeds as follows:

#### Initialisation:

Set $$t = 0$$.

- For $j=1,\cdots , J$, pre-estimate the global parameter $\beta _j^{*}=\tilde{\beta }_{j}$ by maximising $\sum _{i=1}^{n} \log \ f_{\text {ZINB}}\left( X_{ij}; \tilde{\pi }_{j},\exp \left( \tilde{\beta }_{j}+\varvec{B}'_i\varvec{\tilde{\gamma }}\right) ,\tilde{\phi }_j\right)$, assuming homogeneity. The estimators $\tilde{\pi }_{j}$, $\tilde{\gamma }_j$, and $\tilde{\phi }_j$ are also obtained.
- Initialise $$p_k^{(0)} = \frac{1}{K},k = 1, \dots , K$$, $$\gamma _{jk}^{(0)}=\tilde{\gamma }_{j}$$, and $$\phi _j^{(0)}=\tilde{\phi }_j$$.
- Initialise $\beta _{jk}^{(0)} = \tilde{\beta }_{j}\left( 1+0.01 \left( k-1\right) \right)$ and $\pi _{jk}^{(0)} = \tilde{\pi }^{(0)}_j \left( 1+0.01\left( k-1\right) \right) ,k = 1,\dots ,K$, by shifting $\tilde{\beta }_{j}$ and $\tilde{\pi }^{(0)}_j$ to avoid equivalent posterior probabilities at the first iteration.

#### E-step:

Update $t = t+1$. The conditional expectation of $l_c(\varvec{\theta })$ with respect to $\varvec{\theta }^{(t-1)}$ is

4 $$\begin{aligned} E_{\varvec{\theta }^{(t-1)}}(l_c(\varvec{\theta }))= & {} \sum _{k=1}^{K} \sum _{i=1}^{n} \hat{z}_{ik}^{(t)} \left[ \log p_{k}+\sum _{j=1}^{J} \log f_{\text {ZINB}}\left( X_{ij}; \pi _{jk},\exp \left( \beta _{jk}+\varvec{B}'_i\varvec{\gamma }\right) ,\phi _j\right) \right] \nonumber \\{} & {} -\lambda \sum _{j=1}^{J}\sum _{k=1}^{K}\left| \beta _{jk}-\beta _j^{*} \right| , \end{aligned}$$

where $$\hat{z}_{ik}^{(t)} = E_{\varvec{\theta }^{(t-1)}}(z_{ik})= \frac{p_k^{(t-1)}\prod _{j=1}^{J}f_{\text {ZINB}}\left( X_{ij}; \pi _{jk}^{(t-1)},\exp \left( \beta _{jk}^{(t-1)}+\varvec{B}'_i\varvec{\gamma }^{(t-1)}\right) ,\phi _j^{(t-1)}\right) }{\sum _{k=1}^{K}p_k^{(t-1)}\prod _{j=1}^{J}f_{\text {ZINB}}\left( X_{ij}; \pi _{jk}^{(t-1)},\exp \left( \beta _{jk}^{(t-1)}+\varvec{B}'_i\varvec{\gamma }^{(t-1)}\right) ,\phi _j^{(t-1)}\right) }.$$

#### M-step:

Optimise $E_{\varvec{\theta }^{(t-1)}}(l_c(\varvec{\theta }))$ with respect to $\varvec{\theta }$. First, introduce the unobserved indicators $m_{ijk}$’s where $m_{ijk} = 1$ if the value of $X_{ij}$ is produced by dropouts, and $m_{ijk} = 0$ if the value exhibits the true expression level and follows a negative binomial distribution. Then, for $k = 1,\dots ,K$, conduct the following steps sequentially:

- Compute $$p_k^{(t)} = \frac{1}{n} \sum _{i=1}^{n}\hat{z}_{ik}^{(t)}$$.
- Compute $\hat{m}_{ijk}^{(t)} = \frac{\pi _{jk}^{(t-1)}}{\pi _{jk}^{(t-1)}+\left( 1-\pi _{jk}^{(t-1)}\right) \left( \frac{\phi _j^{(t-1)}}{\exp \left( \beta _{jk}^{(t-1)}+\varvec{B}'_i\varvec{\gamma }^{(t-1)}\right) +\phi _j^{(t-1)}}\right) ^{\phi _{j}^{(t-1)}}}$ if $X_{ij} = 0$, and $\hat{m}_{ijk}^{(t)} = 0$, otherwise.
- Compute $$\pi _{jk}^{(t)}= \frac{\sum _{i=1}^{n}\hat{z}_{ik}^{(t)} \hat{m}_{ijk}^{(t)}}{\sum _{i=1}^{n}\hat{z}_{ik}^{(t)}},j = 1,\dots ,J$$.
- Optimise $\phi _{j}^{(t)}=\text {argmax}_{\phi _{j}}\sum _{k=1}^{K} \sum _{i=1}^{n} \hat{z}_{ik}^{(t)} \sum _{j=1}^{J} \left( 1 - \hat{m}_{ijk}^{(t)} \right) \log f_{\text {NB}}\left( X_{ij}; \right.$ $\left. \exp \left( \beta _{jk}^{(t-1)}+\varvec{B}'_i\varvec{\gamma }^{(t-1)}\right) ,\phi _j\right)$ using the Broyden-Fletcher-Goldfarb-Shanno (BFGS) quasi-Newton method for $j = 1,\dots , J$.
- Optimise $\gamma _{s}^{(t)}=\text {argmax}_{\gamma _{s}} \sum _{k=1}^{K} \sum _{i=1}^{n} \hat{z}_{ik}^{(t)} \sum _{j=1}^{J} \left( 1 - \hat{m}_{ijk}^{(t)} \right) \log f_{\text {NB}}\left( X_{ij};\right.$ $\left. \exp \left( \beta _{jk}^{(t-1)}+\varvec{B}'_i\varvec{\gamma } \right) ,\phi _j^{(t)}\right)$ using the BFGS quasi-Newton method for $s = 1,\dots , S$.
- Optimise $\beta _{jk}^{(t)}= \text {argmax}_{\beta _{jk}} \sum _{k=1}^{K} \sum _{i=1}^{n} \hat{z}_{ik}^{(t)} \sum _{j=1}^{J} \left( 1 - \hat{m}_{ijk}^{(t)} \right) \log f_{\text {NB}}\left( X_{ij};\right.$ $\left. \exp \left( \beta _{jk}+\varvec{B}'_i\varvec{\gamma }^{(t)} \right) ,\phi _j^{(t)}\right)$ using the iteratively reweighted least squares method for $j = 1,\dots , J$, as presented in Algorithm S1 (Additional file 3: Supplementary Note).

We iterate the $\textrm{E}$ and $\textrm{M}$ steps until convergence, which is concluded in our numerical study if $\left\| \varvec{\theta }^{(t+1)}-\varvec{\theta }^{(t)}\right\| _{\infty }<10^{-3}$. Convergence is achieved in all of our numerical examples with a moderate number of iterations. In the proposed algorithm, as the closed-form solutions of parameters $\varvec{\phi }$ and $\varvec{\gamma }$ are not available, the BFGS quasi-Newton method is adopted for optimising. This quasi-Newton algorithm has fewer constraints on the convexity of the target function. The BFGS quasi-Newton method has been widely adopted and shown to have satisfactory performance in published studies [56, 57]. We have followed these studies and used the R function “optim” to realise the BFGS quasi-Newton method. The tuning parameter $\lambda$ is determined by the Bayesian Information Criterion (BIC), which is commonly adopted in published studies. Specifically, we consider a candidate set of $\lambda$ with a length of $M = 10$ constructed as $\lambda ^{(i)} = \lambda ^{(1)} + \left( \lambda ^{(M)}-\lambda ^{(1)} \right) (i-1), i = 1, \dots , M$ with $\lambda ^{(1)}=0.01$ and $\lambda ^{(M)}=20$. As in perhaps all studies, the choice of the candidate tuning parameters cannot be fully objective. The adopted strategy has been very common in published studies [6, 21]. Such a choice of candidate values has led to satisfactory performance in our numerical studies. In data analysis, a more refined search can be obtained by expanding the range of candidate parameters, and a reduced range of candidates can help improve the computation efficiency. For each of the candidate values of $\lambda$ and its corresponding estimator $\hat{\varvec{\theta }}$, $\text {BIC} = -2 l (\hat{\varvec{\theta }}) + \log (n) d,$ where $l (\hat{\varvec{\theta }})$ is the log-likelihood in (2), $d = (K-1) + J + S + 2KJ -q$ is the effective number of parameters, and *q* is the number of $\hat{\beta }_{jk}$’s that are shrunken to the global mean. The $\lambda$ value with the lowest BIC is then chosen.

### Competing methods

CIDR, SC3, Seurat, scDeepCluster, MNN-Graph, MNN-Kmeans, RZiMM, snbClust, sparseKmeans, M3Drop, and NBDrop are considered as competing methods. Among these, CIDR [13] is an imputation-based clustering method that handles dropout in scRNA-seq data analysis. SC3 [3] is a popular pipeline for scRNA-seq data, using combined clustering methods. Seurat [24] performs graph-based clustering on the projected space from PCA. scDeepCluster [5] is a deep learning clustering method that integrates the ZINB model with clustering loss. MNN-Graph and MNN-Kmeans are two multi-staged approaches that first perform dimension reduction and correct for batch effects using the MNN approach [9], followed by Graph-based [25] and K-means clustering, respectively. RZiMM [6] develops a zero-inflated NB model with binary subgroup indicators for hard clustering analysis, using a zero-inflated strategy for adjusting for dropouts and penalisation on the pairwise differences of cluster effects for gene selection. snbClust [22] proposes a negative binomial mixture model for clustering analysis as well as a penalty for gene selection. sparseKmeans [26] is a modified K-means algorithm that can also conduct gene selection. M3Drop and NBDrop [19] are two methods that take advantage of the prevalence of zeros in scRNA-seq data to identify features. For the methods with continuous data assumptions, we conduct a transformation of the original count measures following published studies. Specifically, for sparseKmeans, we transform the raw data into CPM by the *edgeR* package following [22], and global-scaling log-transformed normalisation is adopted for Seurat and two MNN-based method. For Seurat that cannot give a specific number of clusters, we run multiple times with the “resolution” parameters tuned from 0.5 to 1.5 by an increase of 0.1 and record the best ARI value as in [58]. CIDR, SC3, and Seurat are implemented using R package *CIDR*, *SC3*, and *Seurat*, respectively. For the two MNN-based methods, fastMNN in R package *batchlor* is first conducted to adjust for batch effects, and graph-based and K-means clustering are then conducted using R package *igraph* and *stats*, respectively. scDeepCluster, RZiMM, and snbClust are implemented using codes from https://github.com/ttgump/scDeepCluster, https://github.com/SkadiEye/RZiMM and https://github.com/YujiaLi1994/snbClust, respectively. SparseKmeans is implemented using R package *sparcl*. M3Drop and NBDrop are conducted with the functions M3DropFeatureSelection and NBumiFeatureSelectionCombinedDrop in R package *M3Drop*, respectively. For each competing method, we adopt the running settings and convergence criterion suggested by the corresponding package or code.

### Data simulation

We consider the following settings. (a) Sample size $n = 300$, number of genes $J = 1000$, percentage of differentially expressed genes $=5\%$, and number of clusters $K = 3$. (b) The baseline parameters are set as follows. We first download the read count matrix of the 3005 cells profiled by [23], genes with low expressions (<10 reads in <20 cells) are excluded, resulting in a total of 2563 genes. Using the expressions of these 2563 genes, we compute the maximum likelihood estimates (MLEs) of the mean and dispersion parameters under the ZINB model. Then, the baseline expression level $\hat{\varvec{\mu }} = \left( \mu _1,\dots ,\mu _J \right) '$ and dispersion $\hat{\varvec{\phi }} = \left( \phi _1,\dots ,\phi _J \right) '$ are obtained by sampling *J* values from the MLEs. (c) Three settings of dropout levels are considered, where the dropout rates $\pi _{jk}$’s are sampled from three different uniform distributions *U*(0.0, 0.3), *U*(0.3, 0.6), and *U*(0.6, 0.9), resulting in low (about 5%), medium (about 45%), and high (about 75%) level of dropout rates, respectively. Here, for each *j*, we sample *K* different $\pi _{jk}$’s from the assumed distribution and $\pi _{jk} \ne \pi _{jk'}$ when $k \ne k'$. (d) Consider two settings for batch effects: $\varvec{\gamma } =(\gamma _1,\gamma _2)'=(0.1,0.2)'$ and $(0.1,0.4)'$ with the number of batches $S = 2$ to account for various levels of batch differences. (e) Consider both balanced and imbalanced cell types, with the mixing proportions $\varvec{p} = (\frac{1}{3},\frac{1}{3},\frac{1}{3})'$ and $(\frac{1}{2},\frac{1}{3},\frac{1}{6})'$, respectively. (f) Set $\beta _{jk} = \log (\hat{\mu }_j\times 2^{\Delta \delta _{jk}})$, where the pattern $\left( \delta _{j 1}, \delta _{j 2}, \delta _{j 3}\right)$ is randomly set to be $(-1,0,1),(1,1,0)$, or (0, 1, 1) for informative genes, and (0, 0, 0) for non-informative genes. Consider three values of $\Delta$, namely 0.8, 1.2, and 1.6, to simulate low, medium, and high levels of biological differences among cell types. Under the aforementioned settings, we first determine the cell type *k* of each cell following a multinomial distribution with probability $\varvec{p}$. For simplicity, we assume a balanced batch distribution where $\varvec{B}_i = \left( 1,0\right) '$ for $i = 1,\dots ,\frac{n}{2}$, and $\varvec{B}_i = (0,1)'$ for $i = \frac{n}{2}+1,\dots ,n$. Then, given the cell type *k* and $\varvec{B}_i$, we generate $X_{ij}$ from $\text {ZINB}\left( \text {exp}\left( \beta _{jk}+\varvec{B}'_i\varvec{\gamma }\right) ;\hat{\phi }_j,\pi _{jk}\right)$.

Beyond the above scenarios with $$n=300$$ and $$K=3$$, we conduct additional simulation under scenarios with various numbers of *n* and *K*. Specifically, we consider $$\varvec{\gamma } = (0.1,0.2)',\Delta = 1.2$$, a balanced sample distribution design, and three levels of dropouts. Then, we first set $$K = 4, 5, 8$$, and 10 with increasing sample sizes, which is common in practical data. In particular, we set $$n=600$$ and informative gene patterns as $$\left( \delta _{j1},\delta _{j2},\delta _{j3},\delta _{j4}\right) \in \left\{ (-1,1,0,1),(1,-1,1,0),(1,1,-1,-1) \right\}$$ for $$K=4$$, $$n=750$$ and $$(\delta _{j1},\delta _{j2},\delta _{j3},\delta _{j4},\delta _{j5}) \in \left\{ (-1,-1,1,0,1),(-1,1,1,1,0),(0,1,-1,-1,-1) \right\}$$ for $$K=5$$, $$n= 1200$$ and $$(\delta _{j1},\delta _{j2},\delta _{j3},\delta _{j4},\delta _{j5}, \delta _{j6},\delta _{j7},\delta _{j8}) \in \{( 1,1,1,-1,-1,-1,0,0 ),$$ $$(-1,-1,0,1,1,-1,1,1),(1,-1,0,1,1,-1,-1,0), (0,1,0,-1,0,-1,-1,-1) \}$$ for $$K = 8$$, and $$n =1500$$ and $$(\delta _{j1},\delta _{j2},\delta _{j3},\delta _{j4},\delta _{j5}, \delta _{j6},\delta _{j7},\delta _{j8}, \delta _{j9},\delta _{j10}) \in \{(-1,1,1,0,-1,-1,$$ $$-1,0,0,1), ( -1,1,-1,1,0,1,1,0,0,1 ), (-1,-1,0, 1,1 ,-1,-1,1,-1,-1), (-1,-1,0,$$ $$1,1,1,0,1,1,1) \}$$ for $$K = 10$$. Second, for the medium dropout rate with $$\pi _{jk} \sim U(0.3,0.6)$$ and $$10\%$$ differentially expressed genes, we further set $$K = 3, 4, 5, 6,$$ and 8 with a fixed total sample size $$n=600$$. In particular, the informative gene patterns for $$K=3,4,5,$$ and 8 are the same as above, and the pattern for $$K=6$$ is set as $$\left( \delta _{j1},\delta _{j2},\delta _{j3},\delta _{j4},\delta _{j5}, \delta _{j6} \right) \in \{ (1,1,-1,-1,0,0),$$ $$(-1,0,1,-1,1,1),(1,0,1,-1,-1,0), (0,0,-1,-1,-1,-1) \}$$.

In the above simulations, we assume that the dropout rate $\pi _{jk}$ of the $j^{\text {th}}$ gene varies across clusters. We additionally consider the scenarios under which dropout rate is independent of clustering with $\pi _{j1}=\cdots =\pi _{jK}\triangleq \pi _j$. Specifically, we consider $n=300$, $K=3$, $10\%$ differentially expressed genes, $\varvec{\gamma } = (0.1,0.2)', \Delta = 1.2$, a balanced sample distribution design, and three levels of dropout rates: about 30%, 50%, and 70% with $\pi _{j} \sim U(0.2,0.4),~ U(0.4,0.6)$ and *U*(0.6, 0.8), respectively.

### Evaluation metrics

For data with known cell-type labels, we use ARI to compare clustering performance of different algorithms. Specifically, for the true and estimated cluster assignment $\mathcal {A}$ and $\hat{\mathcal {A}}$, define $a=$“the number of cell pairs that are assigned to the same cell types with both $\mathcal {A}$ and $\hat{\mathcal {A}}$”, $b=$“the number of cell pairs that are assigned to different cell types with both $\mathcal {A}$ and $\hat{\mathcal {A}}$”, $c=$“the number of cell types that are assigned to the same cell types with $\mathcal {A}$ but to different cell types with $\hat{\mathcal {A}}$”, and $d=$”the number of cell types that are assigned to the same cell types with $\hat{\mathcal {A}}$ but to different cell types with $\mathcal {A}$”. Then, the ARI value is defined as:

$$\begin{aligned} \textrm{ARI}=\frac{\left( \begin{array}{l} n \\ 2 \end{array}\right) (a+d)-[(a+b)(a+c)+(c+d)(b+d)]}{\left( \begin{array}{l} n \\ 2 \end{array}\right) -[(a+b)(a+c)+(c+d)(b+d)]}, \end{aligned}$$

with range $[-1,1]$, where a larger value indicates a higher clustering accuracy.

For simulated data with known significant genes, to evaluate gene selection performance, we adopt $\text {Recall} = \frac{\text {TP}}{\text {TP}+\text {FN}}$, $\text {Precision} = \frac{\text {TP}}{\text {TP}+\text {FP}}$, and $\text {F1} = \frac{2\cdot \text {Precison}\cdot \text {Recall}}{\text {Precison}+ \text {Recall}}$, where TP, FP, and FN are the numbers of true positives, false positives, and false negatives, respectively. Recall, Precision, and F1 range from 0 to 1, with a higher value indicating better gene selection performance.

### Real scRNA-seq datasets

In the mouse embryonic stem cell dataset, the transcriptome of 704 mouse embryonic stem cells was sequenced across three culture conditions (lif, 2i, and a2i), using the Fluidigm C1 microfluidics cell capture platform followed by illumina sequencing [33]. Following the published studies [18], we only consider the cells in the second and third batches wherein all three culture types were collected for the experiments.

The human lung cell dataset was collected from three lung adenocarcinoma cell lines HCC827, H1975, and H2228 on three different platforms with CELseq2, 10x Chromium, and Drop-seq protocols [34], respectively.

The mouse uterus, mouse liver, and mouse mammary gland datasets were processed on the Microwell-seq platform from the Mouse Cell Atlas project [35]. We consider the count matrix of single cells from the uterus, liver, and mammary gland tissues with the corresponding cellular component annotations. Following [59], we remove cells expressing $<250$ genes, genes expressed in $<50$ cells, and cell types representing $<3\%$ of the total population in the tissue.

### Supplementary information

**Additional file 1:** **Supplementary Figs S1-S13.**

**Additional file 2:** **Supplementary Tables S1-S26.**

**Additional file 3.** Supplementary Note. IRLS Algorithm for $\beta_{jk}^{(t)}$ Optimisation.

**Additional file 4.** Review history.

## Data Availability

The scRNA-seq datasets supporting this study are publicly available. The mouse embryonic stem cell dataset is available at http://www.ebi.ac.uk/teichmann-srv/espresso. The human lung cell dataset is available at https://github.com/LuyiTian/sc_mixology [60]. The mouse uterus cell, mouse liver cell, and mouse mammary gland cell datasets are available at https://figshare.com/articles/dataset/MCA_DGE_Data/5435866 [61]. The source code supports the results provided in this paper is available on GitHub : https://github.com/mengyunwu2020/ZINBMM [62] and Zenodo: https://doi.org/10.5281/zenodo.7804487 [63]. Both repositories are released under the GPL-2.0 license.
