# Supplementary material for: ZINBMM: a general mixture model for simultaneous clustering and gene selection using single-cell transcriptomic data
Source: Genome Biol. 2023 Sep 11;24:208. doi: 10.1186/s13059-023-03046-0 (PMC10496184; doi:10.1186/s13059-023-03046-0)
Supplement: Supplementary file 1 — Additional file 1: Supplementary Figs S1-S13. [file 13059_2023_3046_MOESM1_ESM.pdf]

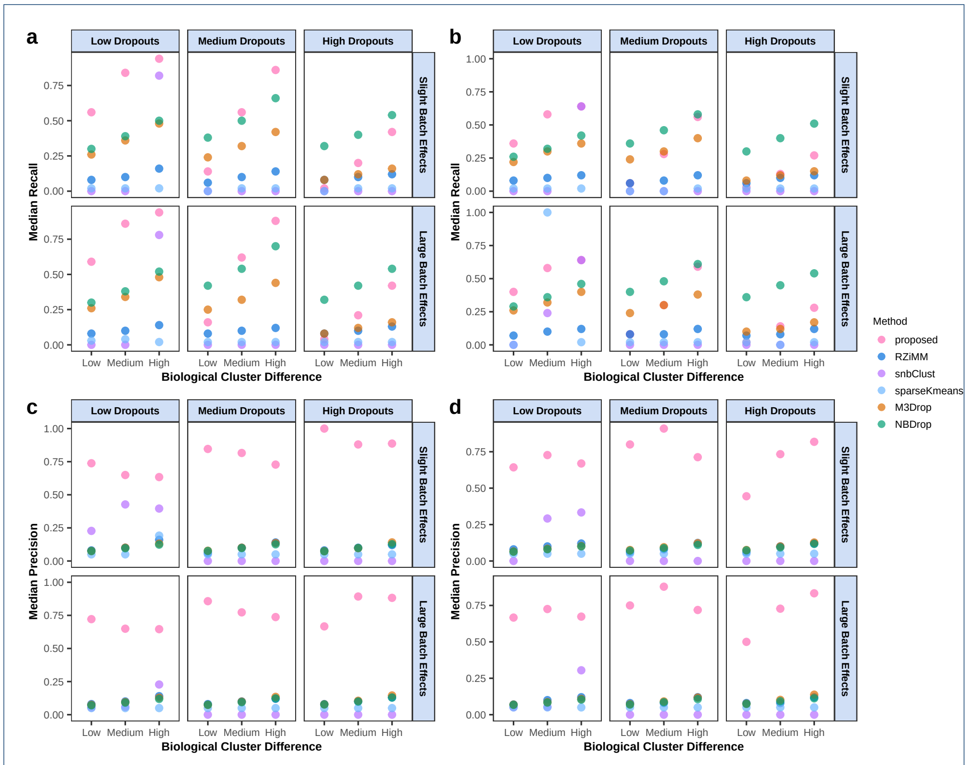

Fig. S1 Simulation results with $K = 3$. Plots of the median Recall under (a) a balanced sample distribution, and (b) an imbalanced sample distribution, as well as median Precision under (c) a balanced sample distribution, and (d) an imbalanced sample distribution over 100 replicates. Different colours represent different methods.

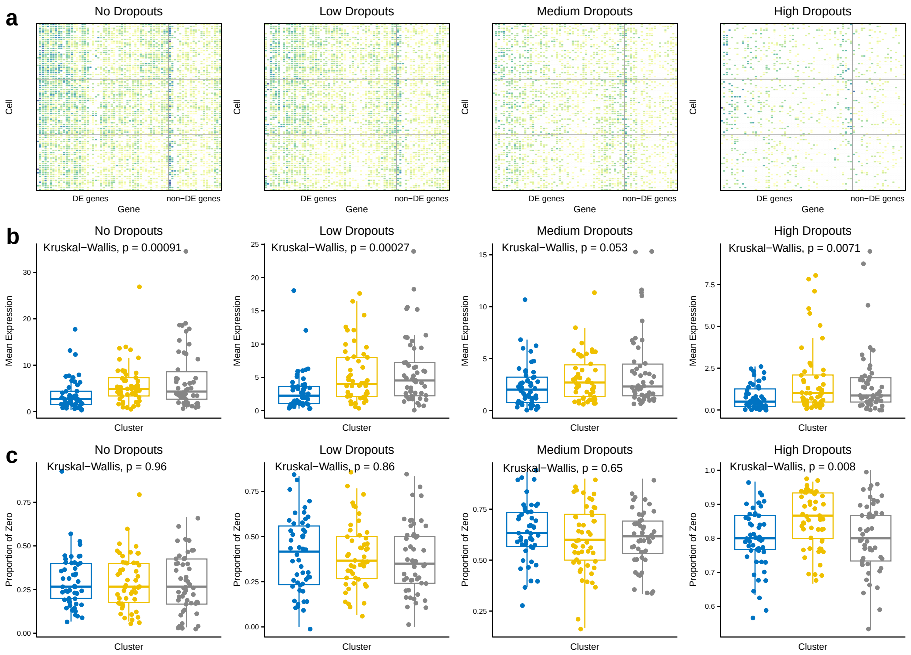

Fig. S2 (a) Heatmaps of expressions of the 50 biologically informative genes and 20 non-informative genes, (b) boxplots of mean expressions of the 50 biologically informative genes, and (c) boxplots of the proportions of zero observations, under the simulation settings with the same biological difference across clusters and batch effects and different levels of dropout rates. The three clusters and two types of genes (informative and non-informative) are separated by the horizontal and vertical lines, respectively, and p value is computed from the Kruskal-Wallis test.

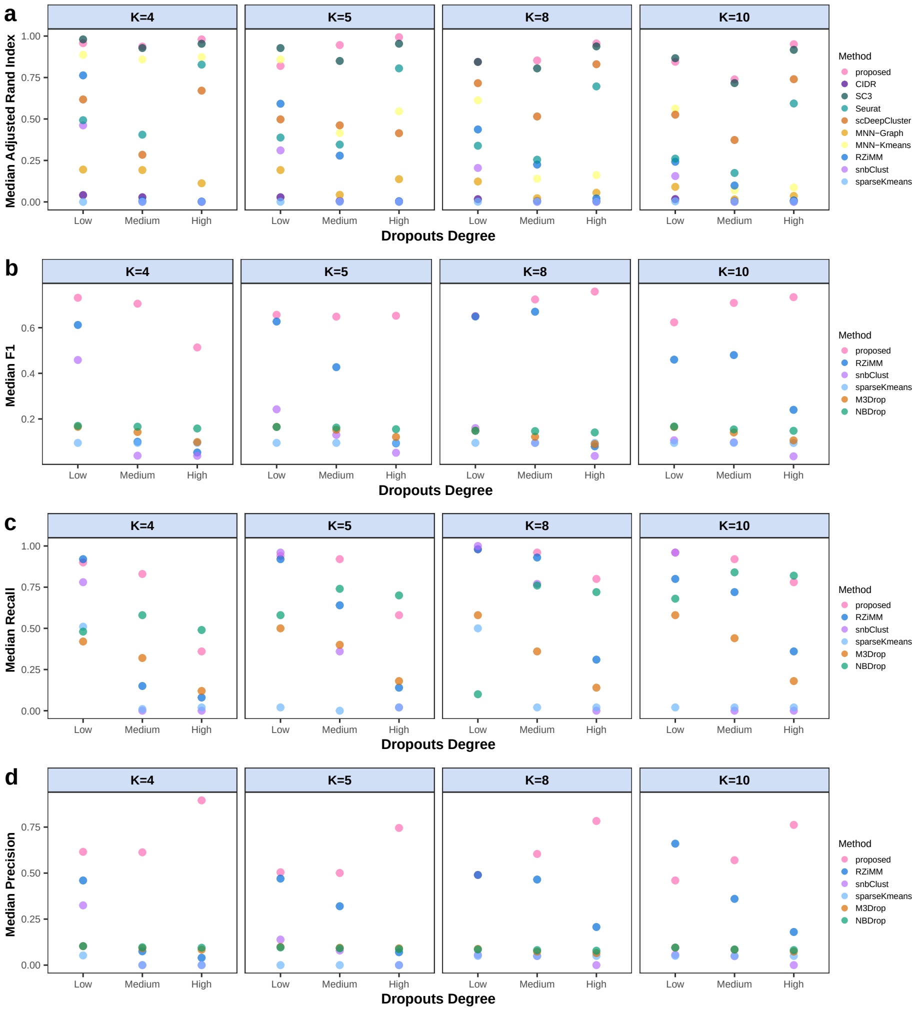

Fig. S3 Simulation results with $K = 4, 5, 8$, and 10. Plots of the (a) median ARI, (b) median F1, (c) median Recall, and (d) median Precision over 100 replicates. Different colours represent different methods.

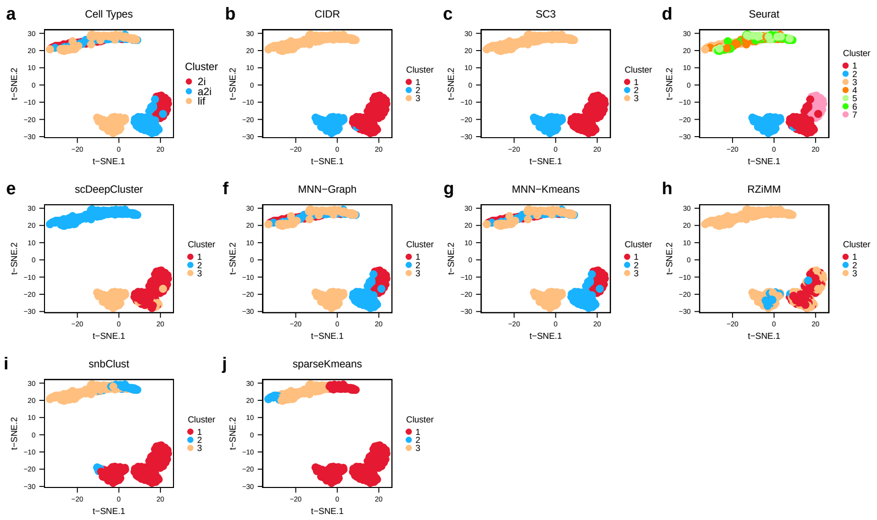

Fig. S4 Two-dimensional t-SNE projection of cells for the mouse embryonic stem cell dataset. The cells are coloured by (a) the annotated cell types, and clustering results identified using (b) CIDR, (c) SC3, (d) Seurat, (e) scDeepCluster, (f) MNN-Graph, (g) MNN-Kmeans, (h) RZiMM, (i) snbClust, and (j) sparseKmeans.

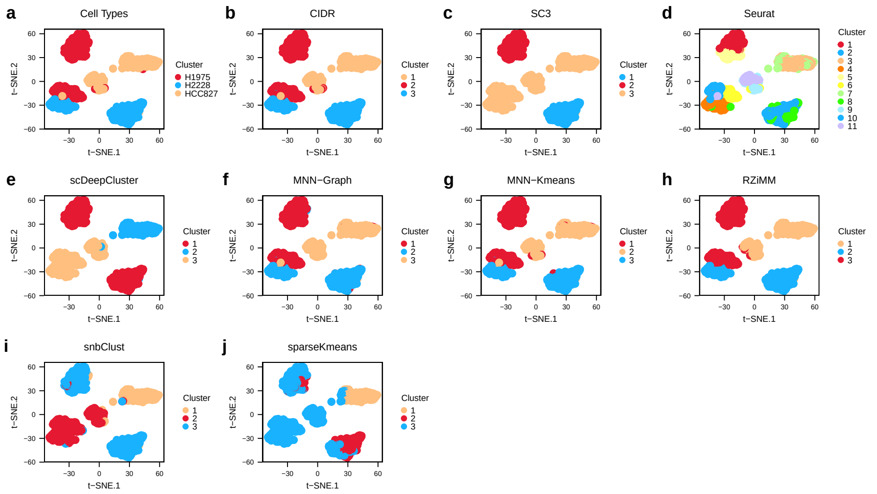

Fig. S5 Two-dimensional t-SNE projection of cells for human lung cell dataset. The cells are coloured by (a) the annotated cell types, and clustering results identified using (b) CIDR, (c) SC3, (d) Seurat, (e) scDeepCluster, (f) MNN-Graph, (g) MNN-Kmeans, (h) RZiMM, (i) snbClust, and (j) sparseKmeans.

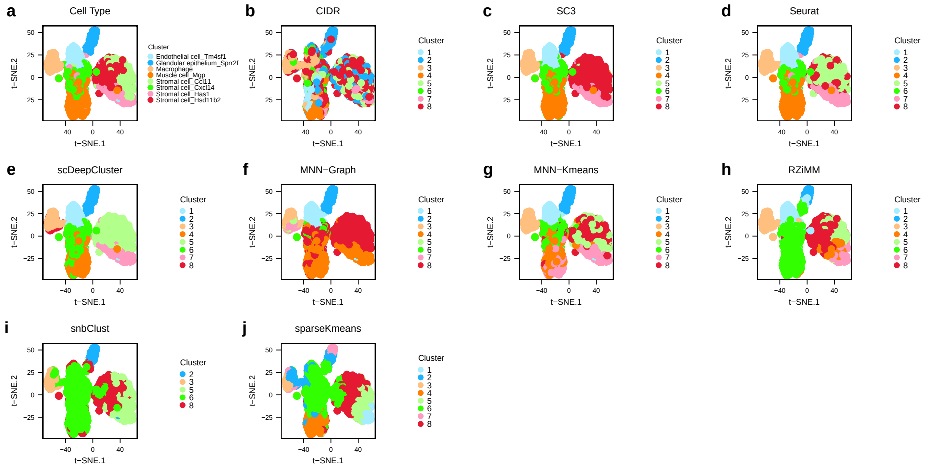

Fig. S6 Two-dimensional t-SNE projection of cells for mouse uterus cell dataset. The cells are coloured by (a) the annotated cell types, and clustering results identified using (b) CIDR, (c) SC3, (d) Seurat, (e) scDeepCluster, (f) MNN-Graph, (g) MNN-Kmeans, (h) RZiMM, (i) snbClust, and (j) sparseKmeans.

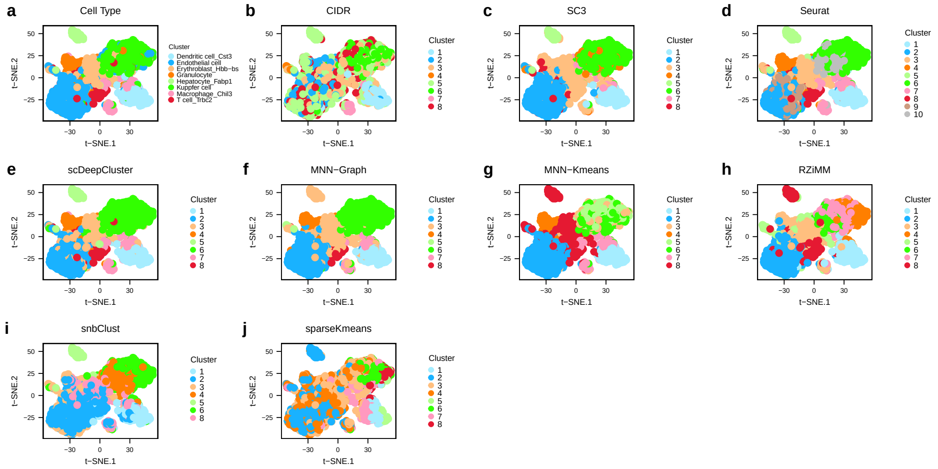

Fig. S7 Two-dimensional t-SNE projection of cells for mouse liver cell dataset. The cells are coloured by (a) the annotated cell types, and clustering results identified using (b) CIDR, (c) SC3, (d) Seurat, (e) scDeepCluster, (f) MNN-Graph, (g) MNN-Kmeans, (h) RZiMM, (i) snbClust, and (j) sparseKmeans.

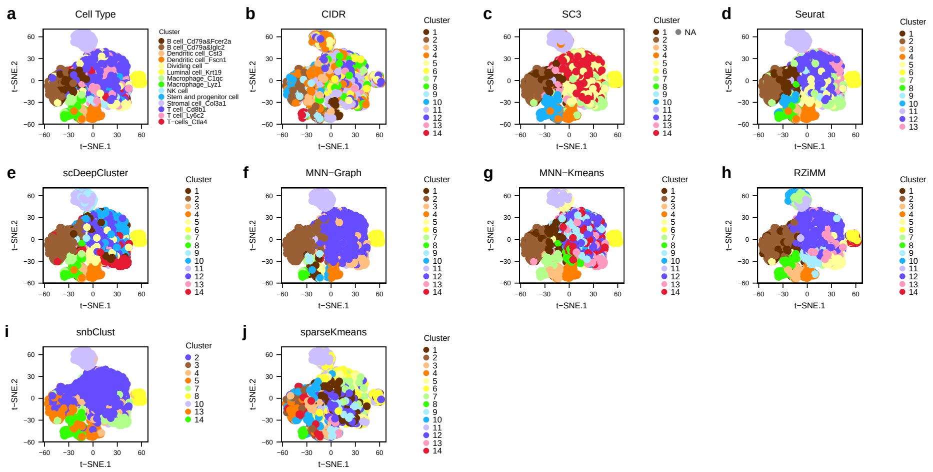

Fig. S8 Two-dimensional t-SNE projection of cells for mouse mammary gland cell dataset. The cells are coloured by (a) the annotated cell types, and clustering results identified using (b) CIDR, (c) SC3, (d) Seurat, (e) scDeepCluster, (f) MNN-Graph, (g) MNN-Kmeans, (h) RZiMM, (i) snbClust, and (j) sparseKmeans.

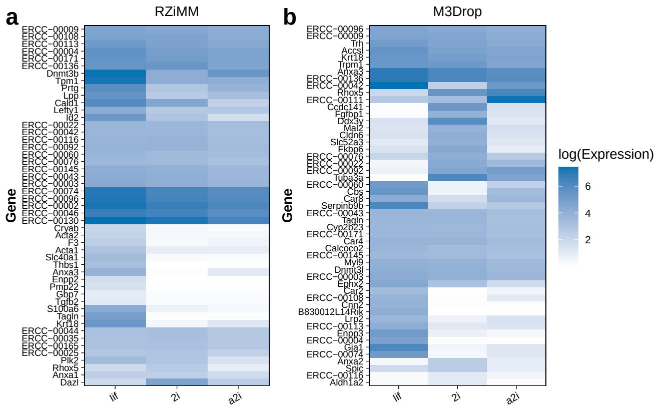

Fig. S9 Log-transformed expressions of the identified genes among different true cell types for mouse embryonic stem cell dataset: (a) RZiMM and (b) M3Drop.

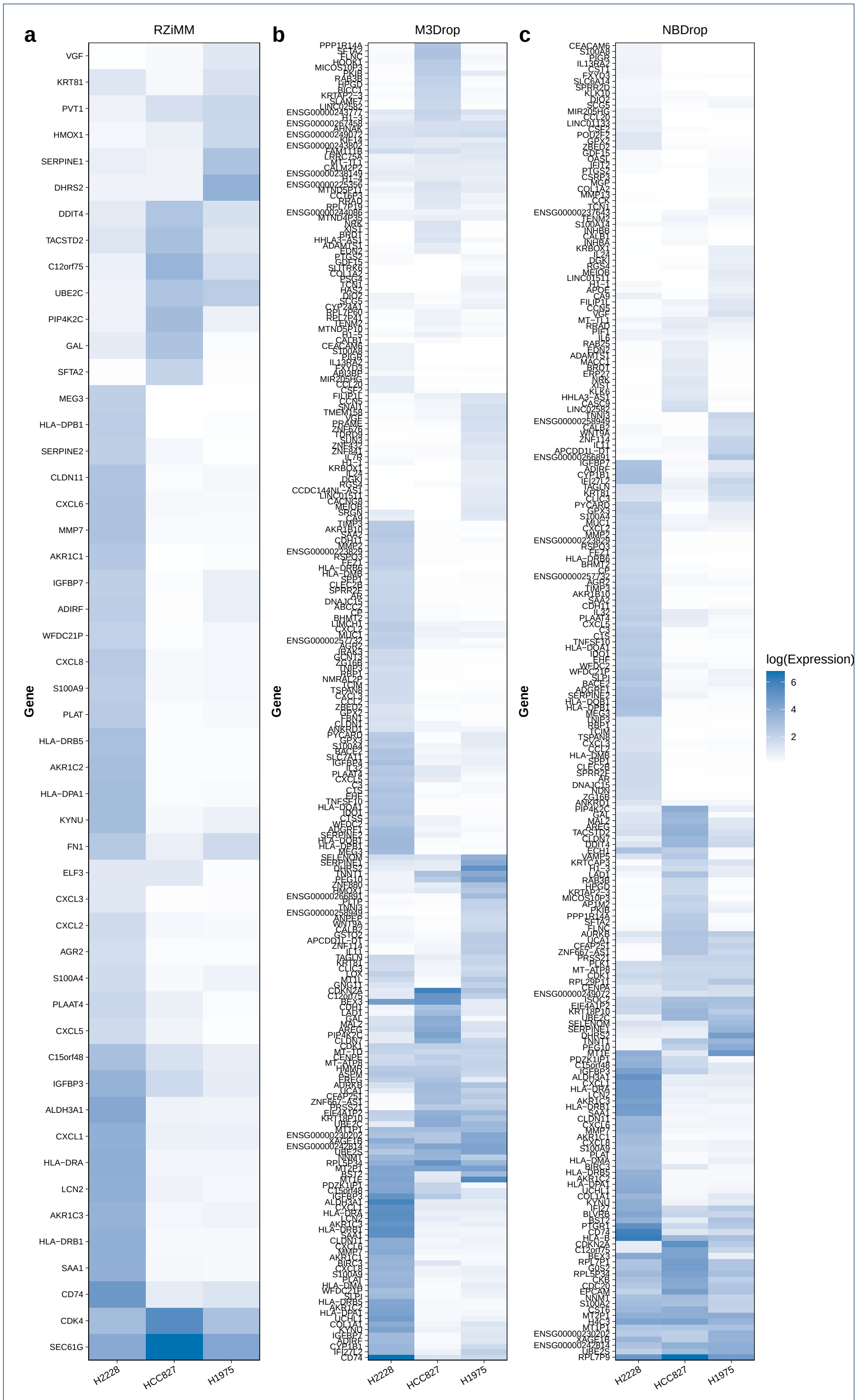

Fig. S10 Log-transformed expressions of the identified genes among different true cell types for human lung cancer cell dataset: (a) RZiMM , (b) M3Drop, and (c) NBDrop.

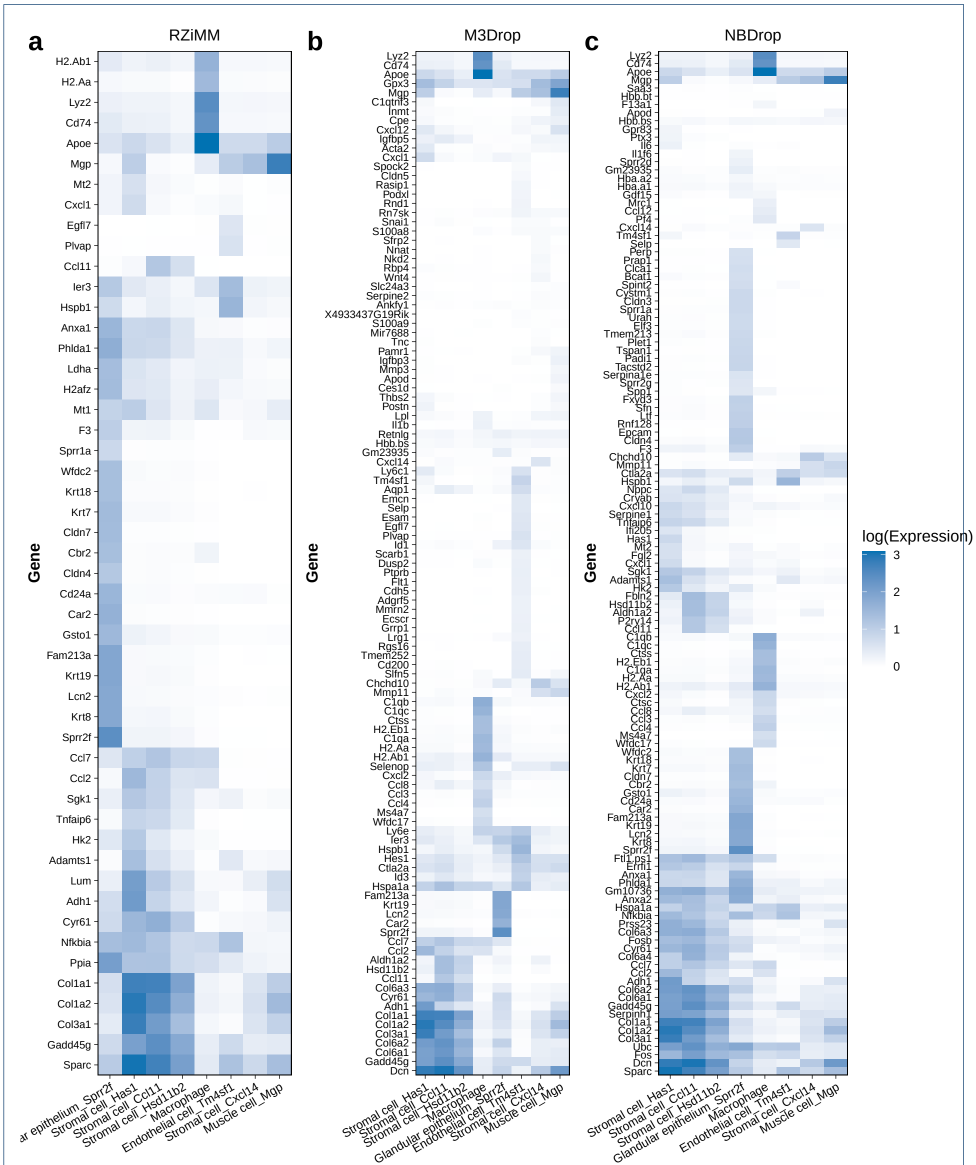

Fig. S11 Log-transformed expressions of the identified genes among different true cell types for mouse uterus cell dataset: (a) RZiMM, (b) M3Drop, and (c) NBDrop.

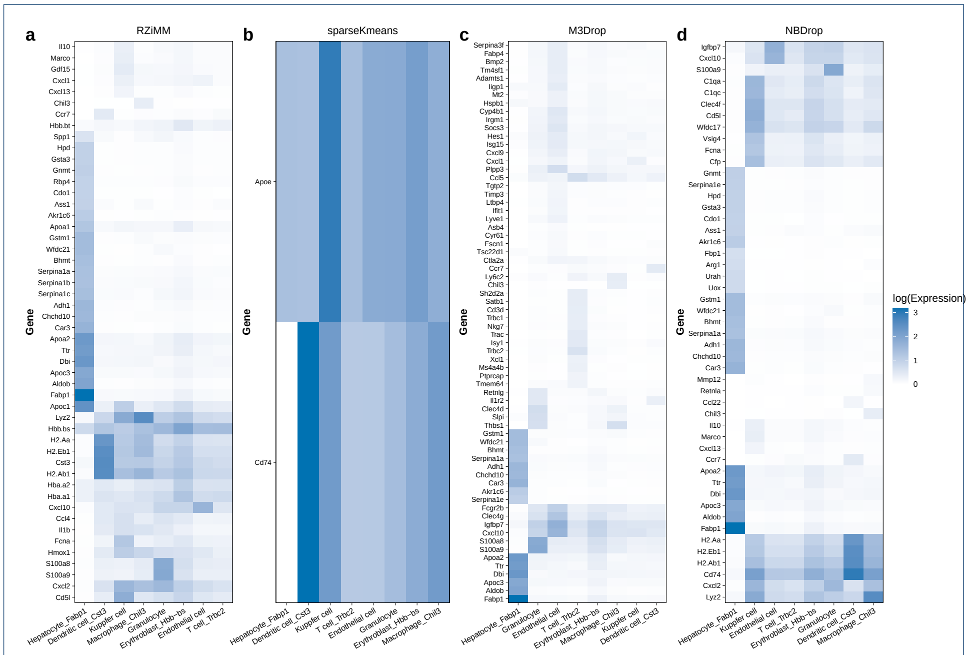

Fig. S12 Log-transformed expressions of the identified genes among different true cell types for mouse liver cell dataset: (a) RZiMM, (b) sparseKmeans, (c) M3Drop, and (d) NBDrop.

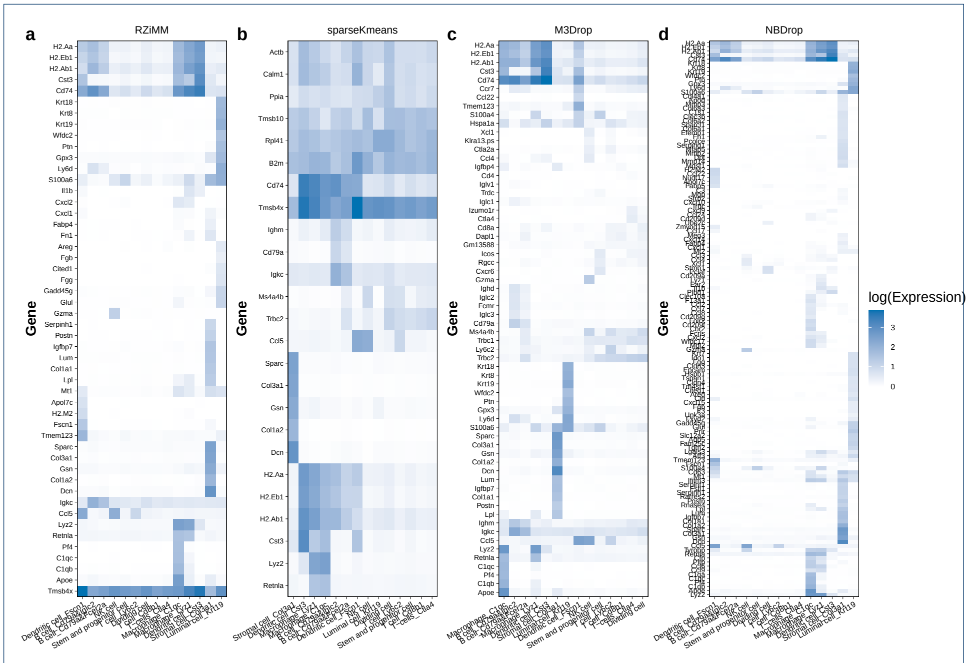

Fig. S13 Log-transformed expressions of the identified genes among different true cell types for mouse mammary gland cell dataset: (a) RZiMM, (b) sparseKmeans, (c) M3Drop, and (d) NBDrop.
