# Supplementary material for: ZINBMM: a general mixture model for simultaneous clustering and gene selection using single-cell transcriptomic data
Source: Genome Biol. 2023 Sep 11;24:208. doi: 10.1186/s13059-023-03046-0 (PMC10496184; doi:10.1186/s13059-023-03046-0)
Supplement: Supplementary file 3 — Additional file 3. Supplementary Note. IRLS Algorithm for $\beta_{jk}^{(t)}$βjk(t) Optimisation. [file 13059_2023_3046_MOESM3_ESM.pdf]

Supplementary Note

IRLS Algorithm for $\beta_{jk}^{(t)}$ Optimisation

Algorithm S1 Optimize $\beta_{jk}^{(t)}$ at step t by IRLS

Input: $X_{\cdot j}, \hat{z}_{\cdot k}^{(t)}, \hat{m}_{\cdot jk}^{(t)}, p_k^{(t)}, \pi_{jk}^{(t)}, \phi_j^{(t)}, \boldsymbol{\gamma}^{(t)}$.

Output: $\beta_{jk}^{(q)}$ as $\beta_{jk}^{(t)}$.

1: **while** $\left|\beta_{jk}^{(q)} - \beta_{jk}^{(q-1)}\right| \geq 10^{-7}$ **do**

2: Update $q = q + 1$.

3: Compute $\omega_{ijk}^{(q+1)} = \frac{\phi_j^{(t)}\left(X_{ij} - \mu_{ijk}^{(q)}\right)}{\mu_{ijk}^{(q)}\left(\mu_{ijk}^{(q)} + \phi_j^{(t)}\right)} \cdot \mu_{ijk}^{(q)} + \frac{-2\phi_j^{(t)} X_{ij} \mu_{ijk}^{(q)} + \phi_j^{(t)}\left(\mu_{ijk}^{(q)}\right)^2 - \left(\phi_j^{(t)}\right)^2 X_{ij}}{\left(\mu_{ijk}^{(q)}\right)^2\left(\mu_{ijk}^{(q)} + \phi_j^{(t)}\right)^2} \cdot \left(\mu_{ijk}^{(q)}\right)^2$,
with $\mu_{ijk}^{(q)} = \exp\left(\beta_{jk}^{(q)} + \boldsymbol{B}_i' \boldsymbol{\gamma}^{(t)}\right)$.

4: Compute $\tau_{ijk}^{(q+1)} = \beta_{jk}^{(q)} - \frac{\phi_j^{(t)}\left(X_{ij} - \mu_{ijk}^{(q)}\right)}{\mu_{ijk}^{(q)}\left(\mu_{ijk}^{(q)} + \phi_j^{(t)}\right)\omega_{ijk}^{(q+1)}} \cdot \mu_{ijk}^{(q)}$.

5: Update $\beta_{jk}^{(q+1)} = \text{argmin}\left[-\frac{1}{2}\sum_{i=1}^n \hat{z}_{ik}^{(t)}\left(1 - \hat{m}_{ijk}^{(t)}\right)\omega_{ijk}^{(q+1)}\left(\tau_{ijk}^{(q+1)} - \beta_{jk}\right)^2 + \lambda\left|\beta_{jk} - \beta_j^*\right|\right]$
with the solution:

$$\beta_{jk}^{(q+1)} = \beta_j^* + \operatorname{sign}\left(\tilde{\beta}_{jk} - \beta_j^*\right) \left[\operatorname{sign}\left(\tilde{\beta}_{jk} - \beta_j^*\right)\left(\frac{\sum_i \hat{z}_{ik}^{(t)}\left(1 - \hat{m}_{ijk}^{(t)}\right) w_{ijk}^{(q+1)} \tau_{ijk}^{(q+1)} + \lambda \operatorname{sign}\left(\tilde{\beta}_{jk} - \beta_j^*\right)}{\sum_i \hat{z}_{ik}^{(t)}\left(1 - \hat{m}_{ijk}^{(t)}\right) w_{ijk}^{(q+1)}} - \beta_j^*\right)\right]_+,$$

where $\tilde{\beta}_{jk} = \frac{\sum_i \hat{z}_{ik}^{(t)}\left(1 - \hat{m}_{ijk}^{(t)}\right) w_{ijk}^{(q+1)} \tau_{ijk}^{(q+1)}}{\sum_i \hat{z}_{ik}^{(t)}\left(1 - \hat{m}_{ijk}^{(t)}\right) w_{ijk}^{(q+1)}}$ and $f_+ = \max\{f, 0\}$.

6: **end while**
