# Supplementary material for: ZINBMM: a general mixture model for simultaneous clustering and gene selection using single-cell transcriptomic data
Source: Genome Biol. 2023 Sep 11;24:208. doi: 10.1186/s13059-023-03046-0 (PMC10496184; doi:10.1186/s13059-023-03046-0)
Supplement: Supplementary file 4 — Additional file 4. Review history. [file 13059_2023_3046_MOESM4_ESM.docx]

Review History

**First round of review**

**Reviewer 1**

**Were you able to assess all statistics in the manuscript, including the appropriateness of statistical tests used?**

**Yes:** looks fine

**Were you able to directly test the methods?**

No

**Comments to author:**

Contribution
This paper proposes a mixture ZINB model ZINBMM to simultaneously implement clustering and gene selection for scRNA-seq data. ZINBMM is able to handle batch effects and dropout in the clustering process.

However, I have the following concerns:

1) the authors do not provide a precise method for selecting genes. They only mentioned "……, \beta_j shrunk to \beta_j^* is considered as an unimportant gene with probably the same expression among all cell types, ……" on page 15. However, the paper does not describe how the "\beta" value is used to select genes.

2) on page 16, the authors wrote "Convergence is achieved in all of our numerical examples with a moderate number of iterations". Adding some numerical results of the program running time would be good.

3) on page 17, the authors first simulated data with K=3 and then simulated data with K = 4 and 5 to mimic the data having a larger number of clusters. However, the difference, there is little difference between these two settings. If the authors want to simulate data with a larger number of clusters, they need to set K to a larger value (e.g., K = 8 or 10).

4) On page 6, the authors need to explain why some evaluation metrics (ARI, F1 values, etc.) are unavailable for some benchmarks.

5) On page 6, The authors should further explain why the methods perform worse when the dropout is moderate.

6) The authors only demonstrate the gene selection results on the real datasets. They should also show the gene selection results on the simulated datasets by comparing the selected genes with the ground truth informative genes.

7) On page 11, Figure 6(b) fails to show the difference between predictive and empirical data distribution clearly.

8) On page10, in Figure 5, are the input to the t-SNE raw counts or normalized data?

9) How many genes are left for each real data after preprocessing? Can the ZINBMM model handle inputs containing more than 10,000 genes?

10) The authors need to provide further explanations of Table S1 - S4. Why can they demonstrate the proposed method is better than other benchmarks

11) The authors should clarify that the log(Expression) shown in Fig 6 and Fig S7-10 are based on the predicted cluster or the ground truth cell type. In Mouse uterus cells dataset, the ARI for the proposed method is around 0.6, which indicates that the predicted clusters and the ground truth cell types are very different. How could a poor clustering provide good gene selection results?

12) The authors should elaborate on how to use BIC to select \lambda for the proposed model.

**Reviewer 2**

**Were you able to assess all statistics in the manuscript, including the appropriateness of statistical tests used?**

No

**Were you able to directly test the methods?**

No

**Comments to author:**

This paper proposes a zero-inflated negative binomial mixture model (ZINBMM) to simultaneously cluster scRNA-seq data in cell types and identify critical genes in each resulting cell type. Identifying critical genes in each cluster is a novel and useful feature of the tool. The model works on the raw counts and considers both batch effects and dropout events without the need of imputation. When evaluated on both simulated and real scRNA-seq datasets, the method outperforms competing methods in most cases, given the number of cell types in a dataset is known beforehand. Although the mothed might be useful in small datasets with few cell types (<=3), I have the following concerns.

Major concerns:

1. More than 1,000 scRNA-seq clustering methods has been developed, many perform well on small datasets with simpler data structures, i.e., containing a small number of cells (<5,000) with few cell types. What the research community urgently need is highly efficient tools that are scalable to datasets containing millions of cells belonging to dozens of cell types. The datasets used to test the proposed tool are quite small (<4,000 cells), and time complexity of the tool was not given and running time data was not presented in comparison with existing tools, in particular fast ones, such as Suerat.

2. The proposed method failed to reasonably identify the number of cell types when the member of cell types is larger than 8. This leads one to question the usefulness of the tool in practice, because in reality we do not know the number of cell types in a dataset.


3. While at least more than 8,868 genes were expressed in the four real datasets, why only the top 1,000 highly variable genes are used in the analysis? The number of genes contributing to cell type formation are likely larger than this number, given that there are at least more than 2,000 transcription factors that mainly determine cell types. Other numbers should be tested for best performance.

4. A highly scalable method scDeepCluster that also uses a ZINB model and was cited in the paper, should be compared with for accuracy and speed.

Minor concerns:

1. Figure 2 only shows the accuracy of clustering results, there is no information about the selection of genes, but the authors stated that "As seen in Fig. 2 (c) and (d), the advantage of the proposed method on gene selection accuracy is strongly evident." Similar overstatements include "… the proposed method is consistently comprehensively superior in terms of both clustering and gene selection accuracy".

**Authors Response**

**Point-by-point responses to the reviewers’ comments:**

Response to Editor
We would like to thank you, the associate editor, and two reviewers for your careful review and insightful comments. Following your suggestions, we have conducted a major revision. Major changes have been highlighted in the manuscript. In addition, we have also made substantial editorial changes to improve the presentation. Please see below for our point-by-point response.

1. Both reviewers request that you demonstrate performance on bigger datasets with higher number of cell types.
Response: In the simulation, we have added the scenarios with a higher number of cell types (K=8 and 10). Moreover, in data analysis, we have conducted an additional analysis on the mammary gland cell dataset from the Mouse Cell Atlas (MCA), which contains 5,154 cells and 14 cell types. Relevant results have been added to the main text and Supplementary Materials. The proposed method is again observed to have satisfactory performance.

2. Referee 2 states that you should compare your method to scDeepCluster.
Response: In both simulation and real data analysis, we have conducted the analysis using the scDeepCluster approach. Relevant results and discussions have also been added.

3. Both reviewers also ask that you provide appropriate metrics for these comparisons. Furthermore, both reviewers raised concerns related to gene selection. As you can see, a revised manuscript should demonstrate the impact of the number of input and selected genes, include simulated data and convince of gene selection accuracy.
Response: In both simulation and real data analysis, we have evaluated the clustering accuracy of each approach using the adjusted Rand index (ARI). Here, ARI’s are computed based on the estimated and true memberships, where each cell in data analysis has been annotated by cell and lineage markers. In addition, in simulation, we have also evaluated gene selection performance using Recall, Precision, and F1, based on the true important simulated genes. These four measures are perhaps the most common measure for evaluating clustering and gene selection accuracy.

In data analysis, as the underlying true model is not available, it is difficult to objectively evaluate the gene selection accuracy. To provide indirect support, we have presented an expression heatmap of the selected genes based on the ground true cell types, and the predictive and empirical data distributions for the selected genes. In addition, we have conducted the literature search and GO enrichment analysis to demonstrate the biological implications of the selected genes for cell type formation.

Following your suggestion, in addition to the analysis with 1,000 input genes, we have also conducted the analysis with 2,000 highly variable genes (HVGs). The comparison results have been provided in Additional file 2: Table S7. It is observed that both the clustering and gene selection results do not significantly depend on the number of input genes. For example, for the mouse embryonic stem cell dataset, the ARI is again equal to 1 with a total of 35 genes selected compared to 33 of 1,000 HVGs. There are 28 overlaps between the two analyses. As the choice of 1,000 HVGs has been widely adopted in many recent scRNA-seq clustering analysis studies, such as Sun et al. (2019), Deng et al. (2019), Chen et al. (2020), and Stassen et al. (2020), we follow this common choice and present the results based on 1,000 HVGs. Discussions have been added in the Section “Discussion and conclusions”.

Reference
[1] Chen, L., Zhai, Y., He, Q., Wang, W., Deng, M.: Integrating deep supervised, self-supervised and unsupervised learning for single-cell rna-seq clustering and annotation. Genes 11(7), 792 (2020).
[2] Deng, Y., Bao, F., Dai, Q., Wu, L.F., Altschuler, S.J.: Scalable analysis of cell-type composition from single-cell transcriptomics using deep recurrent learning. Nature methods 16(4), 311–314 (2019).
[3] Sun, Z., Chen, L., Xin, H., Jiang, Y., Huang, Q., Cillo, A.R., Tabib, T., Kolls, J.K., Bruno, T.C., Lafyatis, R., et al.: A bayesian mixture model for clustering droplet-based single-cell transcriptomic data from population studies. Nature Communications 10(1), 1–10 (2019).
[4] Stassen, S.V., Siu, D.M., Lee, K.C., Ho, J.W., So, H.K., Tsia, K.K.: Parc: ultrafast and accurate clustering of phenotypic data of millions of single cells. Bioinformatics 36(9), 2778–2786 (2020).

Response to Reviewer 1
We would like to thank you, the associate editor, and the other reviewer for your careful review and insightful comments. Following your suggestions, we have conducted a major revision. Major changes have been highlighted in the manuscript. In addition, we have also made substantial editorial changes to improve the presentation. Please see below for our point-by-point response.

1. the authors do not provide a precise method for selecting genes. They only mentioned "……, \beta_j shrunk to \beta_j^* is considered as an unimportant gene with probably the same expression among all cell types, ……" on page 15. However, the paper does not describe how the "\beta" value is used to select genes.
Response: Here, gene j that has all \beta_{j1}, \dots, \beta_{jK} equal to \beta_j^{*} will be identified as an unimportant gene. The important genes, on the other hand, are those with at least one \beta_{jk} different from \beta_j^{*}. Detailed descriptions have been provided in the ‘Methods’ section.

2. on page 16, the authors wrote "Convergence is achieved in all of our numerical examples with a moderate number of iterations". Adding some numerical results of the program running time would be good.
Response: We thank you for this insightful suggestion. We have added the numerical results of the program running time under various simulation settings. Corresponding results have been reported in Table S1 of Additional file 2 and discussed in Section “Testing ZINBMM using simulated data”.

3. on page 17, the authors first simulated data with K=3 and then simulated data with K = 4 and 5 to mimic the data having a larger number of clusters. However, the difference, there is little difference between these two settings. If the authors want to simulate data with a larger number of clusters, they need to set K to a larger value (e.g., K = 8 or 10)
Response: Following your suggestions, we have added simulations with larger true numbers of clusters with K being 8 and 10. Results and discussions have been added and provided in Figure S2 of Additional file 1 and Section “Testing ZINBMM using simulated data”. The superiority of the proposed approach on both clustering and gene selection accuracy is again observed.

4. On page 6, the authors need to explain why some evaluation metrics (ARI, F1 values, etc.) are unavailable for some benchmarks.
Response: Explanations have been added to the Section “Testing ZINBMM using simulated data”.

5. On page 6, The authors should further explain why the methods perform worse when the dropout is moderate.
Response: Further explanations and related references have been added as suggested.

6. The authors only demonstrate the gene selection results on the real datasets. They should also show the gene selection results on the simulated datasets by comparing the selected genes with the ground truth informative genes.
Response: In the simulations, we have adopted Recall, Precision, and F1 to quantify the gene selection performance, by comparing the selected genes with the ground truth informative genes. Detailed definitions have been provided in the Section “Methods”. These three measures are perhaps the most popular choices in published gene selection studies. Relevant results have been presented in Fig. 2 (c) and (d) of the main text, and Fig. S1, Fig. S2 (b), Fig. S2 (c), and Fig. S2 (d) of the Additional file 1. Discussions have also been provided in the Section “Testing ZINBMM using simulated data”.

7. On page 11, Figure 6(b) fails to show the difference between predictive and empirical data distribution clearly.
Response: To enhance the presentation, we have changed the colors and transparencies of the bar plots in Figure 6(b). We would like to point out that because the distributions of the predicted and empirical data fit so well, their differences are difficult to tell apart.

8. On page10, in Figure 5, are the input to the t-SNE raw counts or normalized data?
Response: The inputs are the raw counts which are then projected into the two-dimensional space using t-SNE projection approach (Van der Maaten and Hinton, 2008). Discussions have been added in the Section “Applying ZINBMM to real scRNA-seq datasets”.

9. How many genes are left for each real data after preprocessing? Can the ZINBMM model handle inputs containing more than 10,000 genes?
Response: In our analysis, we have conducted a pre-screening by selecting the top 1,000 highly variable genes (HVGs). In principle, the proposed approach can be directly applied to the original data with more than 10,000 genes. However, considering
that only a small number of genes are potentially associated with the formation of cell types, and that Chen et al. (2020a) and Ciortan and Defrance (2021) have stated:
the screening step could “filter out the genes carrying insufficient information for identifying cell types”, “amplify the underlying information in the retained genes”, and “significantly speed up the model calculation process”, we conduct a prescreening for better performance and more effective complement. Such a choice of 1,000 HVGs has also been adopted in many recent studies for scRNA-seq clustering analyses, such as Sun et al. (2019), Deng et al. (2019), Chen et al. (2020b), and Stassen et al. (2020). Discussions have been added in the Section “Applying ZINBMM to real scRNA-seq datasets”.

10. The authors need to provide further explanations of Table S1 - S4. Why can they demonstrate the proposed method is better than other benchmarks
Response: We would like to note that Tables S1-S4 are only used to present the number of genes identified by different methods and their overlaps, and are not used to demonstrate the superiority of the proposed method. They can provide a general idea of the differences and similarities between the findings with different methods. Similar tables have also been presented in recent scRNA-seq clustering analysis studies, such as Shao et al. (2020) and Gao et al. (2021). Discussions on Table S1 - S4 have been added in the Section “Applying ZINBMM to real scRNA-seq datasets”.

11. The authors should clarify that the log(Expression) shown in Fig 6 and Fig S7-10 are based on the predicted cluster or the ground truth cell type. In Mouse uterus cells dataset, the ARI for the proposed method is around 0.6, which indicates that the predicted clusters and the ground truth cell types are very different. How could a poor clustering provide good gene selection results?
Response: Following your suggestion, we have clarified that the log(Expression) in Fig.6 and Figs. S7-10 is based on the ground truth cell types.

The ARI value lies in [-1,1], with a larger value indicating better clustering performance. For the mouse uterus cell dataset, the ARI value for the proposed method is 0.668, and the corresponding clustering accuracy is about 78%. Thus, true cluster labels are given to almost 75% of the cells. The proposed ZINBMM is able to effectively capture the biological information from these appropriately assigned cells while remaining resistant to the misclassified cells, leading to satisfactory gene selection results. Discussions have been added in the Section “Applying ZINBMM to real scRNA-seq datasets”.

12. The authors should elaborate on how to use BIC to select \lambda for the proposed model.
Response: Following your suggestion, we have added the discussions on how to use BIC to select \lambda for the proposed model in the Section “Methods”.

Reference
[1] Chen, L., Wang, W., Zhai, Y., Deng, M.: Single-cell transcriptome data clustering via multinomial modeling and adaptive fuzzy k-means algorithm. Frontiers in genetics 11, 295 (2020a).
[2] Chen, L., Zhai, Y., He, Q., Wang, W., Deng, M.: Integrating deep supervised, self-supervised and unsupervised learning for single-cell rna-seq clustering and annotation. Genes 11(7), 792 (2020b).
[3] Ciortan, M., Defrance, M.: Contrastive self-supervised clustering of scRNA-seq data. BMC Bioinformatics 22(1), 1–27 (2021).
[4] Deng, Y., Bao, F., Dai, Q., Wu, L.F., Altschuler, S.J.: Scalable analysis of cell-type composition from single-cell transcriptomics using deep recurrent learning. Nature methods 16(4), 311–314 (2019).
[5] Gao, S., Dai, Y., & Rehman, J.: A Bayesian inference transcription factor activity model for the analysis of single-cell transcriptomes. Genome research, 31(7), 1296-1311 (2021).
[6] Sun, Z., Chen, L., Xin, H., Jiang, Y., Huang, Q., Cillo, A.R., Tabib, T., Kolls, J.K., Bruno, T.C., Lafyatis, R., et al.: A bayesian mixture model for clustering droplet-based single-cell transcriptomic data from population studies. Nature Communications 10(1), 1–10 (2019).
[7] Stassen, S.V., Siu, D.M., Lee, K.C., Ho, J.W., So, H.K., Tsia, K.K.: Parc: ultrafast and accurate clustering of phenotypic data of millions of single cells. Bioinformatics 36(9), 2778–2786 (2020).
[8] Shao, X., Liao, J., Lu, X., Xue, R., Ai, N., & Fan, X.: scCATCH: automatic annotation on cell types of clusters from single-cell RNA sequencing data. Iscience, 23(3), 100882. (2020).
[9] Van der Maaten, L., Hinton, G.: Visualizing data using t-SNE. Journal of Machine Learning Research 9(11) (2008).

Response to Reviewer 2
We would like to thank you, the associate editor, and the other reviewer for your careful review and insightful comments. Following your suggestions, we have conducted a major revision. Major changes have been highlighted in the manuscript. In addition, we have also made substantial editorial changes to improve the presentation. Please see below for our point-by-point response.

Major concerns:
1. More than 1,000 scRNA-seq clustering methods has been developed, many perform well on small datasets with simpler data structures, i.e., containing a small number of cells (<5,000) with few cell types. What the research community urgently need is highly efficient tools that are scalable to datasets containing millions of cells belonging to dozens of cell types. The datasets used to test the proposed tool are quite small (<4,000 cells), and time complexity of the tool was not given and running time data was not presented in comparison with existing tools, in particular fast ones, such as Suerat.
Response: Following your suggestion, in data analysis, we have conducted an additional analysis on the mammary gland cell dataset from the Mouse Cell Atlas (MCA), which contains 5,154 cells and 14 cell types. All the five real datasets have been commonly analyzed in recent scRNA-seq clustering analysis studies, such as SiaClust in Jiang et al. (2022), scMAGIC in Zhang et al. (2022), and graph-sc in Ciortan and Defrance (2022). Moreover, in the simulation, we have added the scenarios with a higher number of cell types (K=8 and 10). Relevant results have been added to the main text and Supplementary Materials. The proposed method is again observed to have satisfactory performance.

We have added the Seurat method as the alternative in both simulation and data analysis. For the proposed method and all alternatives, we have added the numerical results of the program running time under various simulation settings. Corresponding results have been reported in Table S1 of Additional file 2 and discussed in Section “Testing ZINBMM using simulated data”.

2. The proposed method failed to reasonably identify the number of cell types when the member of cell types is larger than 8. This leads one to question the usefulness of the tool in practice because in reality we do not know the number of cell types in a dataset.
Response: We thank you for sharing your concerns. In recent scRNA-seq data clustering studies, it is still a widely open problem to determine the number of cell types. As stated by Kiselev et al. (2019), “there is no consensus on the correct method for choosing for choosing k, judgement from the researcher is required.”, and as stated by Sun et al. (2019), “the number of cell types is often considered as a continuum”. In our study, as shown in Table 3, few methods can identify the true numbers of the cell types. Speciﬁcally, SC3, Seurat, and scDeepCluster tend to choose larger numbers of clusters while the optimal numbers decided by gap statistics for two Kmeans-based approaches are small.

In fact, it is observed that the proposed method tends to cluster the cell types with certain similarities into large cell types. For example, three subtypes Cxcl14, Has1, and Hsd11b2 from Stromal are grouped into two subtypes in mouse uterus cells. Two B cell subtypes and three T cell subtypes are clustered into two groups separately in mammary gland cells. This is probably due to that the modified BIC potentially tends to select models with a relatively smaller number of parameters. Here, the modified BIC criterion is adopted, as it has been the most popular in published model-based clustering studies and demonstrated to have satisfactory performance (Mi et al. 2021, Li et al. 2021). If necessary, it is convenient for the proposed method to further cluster cells based on the existing results for the purpose of new subtypes discovery. Additional investigation on the optimal number of clusters is deferred to further studies. Relevant discussions have been added in the Section “Applying ZINBMM to real scRNA-seq data” and the Section “Discussion and conclusion”.

3. While at least more than 8,868 genes were expressed in the four real datasets, why only the top 1,000 highly variable genes are used in the analysis? The number of genes contributing to cell type formation are likely larger than this number, given that there are at least more than 2,000 transcription factors that mainly determine cell types. Other numbers should be tested for best performance.
Response: Following your suggestion, in addition to the analysis with 1,000 input genes, we have also conducted the analysis with 2,000 highly variable genes (HVGs). The comparison results have been provided in Table S7 (Additional File 2). It is observed that both the clustering and gene selection results do not significantly depend on the number of input genes. For example, for the mouse embryonic stem cell dataset, the ARI is again equal to 1 with a total of 35 genes selected compared to 33 of 1,000 HVGs. There are 28 overlaps between the two analyses. As the choice of 1,000 HVGs has been widely adopted in many recent scRNA-seq clustering analysis studies, such as Sun et al. (2019), Deng et al. (2019), Chen et al. (2020), and Stassen et al. (2020), we follow this common choice and present the results based on 1,000 HVGs. Discussions have been added in the Section “Applying ZINBMM to real scRNA-seq data” and the Section “Discussion and conclusion”.

4. A highly scalable method scDeepCluster that also uses a ZINB model and was cited in the paper, should be compared with for accuracy and speed.
Response: Following your suggestion, in both simulation and real data analysis, we have conducted the analysis using the scDeepCluster approach. Relevant results, including clustering performance and computational efficiency, and discussions have also been added.

Minor concerns:
1. Figure 2 only shows the accuracy of clustering results, there is no information about the selection of genes, but the authors stated that "As seen in Fig. 2 (c) and (d), the advantage of the proposed method on gene selection accuracy is strongly evident." Similar overstatements include "… the proposed method is consistently comprehensively superior in terms of both clustering and gene selection accuracy".
Response: In the simulations, we have adopted Recall, Precision, and F1 to quantify the gene selection performance, by comparing the selected genes with the ground truth informative genes. Detailed definitions have been provided in the Section “Methods”. Summarized F1 results under the scenarios with K=3 have been presented in Fig. 2 (c) and (d) of the main text, and the remaining results have been presented in Fig. S1, Fig. S2 (b), Fig. S2 (c), and Fig. S2 (d) of the Additional file 1. Discussions have also been provided in the Section “Testing ZINBMM using simulated data”.

Reference
[1] Chen, L., Zhai, Y., He, Q., Wang, W., Deng, M.: Integrating deep supervised, self-supervised and unsupervised learning for single-cell rna-seq clustering and annotation. Genes 11(7), 792 (2020).
[2] Ciortan, M., & Defrance, M.: GNN-based embedding for clustering scRNA-seq data. Bioinformatics, 38(4), 1037-1044. (2022).
[3] Deng, Y., Bao, F., Dai, Q., Wu, L.F., Altschuler, S.J.: Scalable analysis of cell-type composition from single-cell transcriptomics using deep recurrent learning. Nature methods 16(4), 311–314 (2019).
[4] Jiang, H., Huang, Y., & Li, Q.: Spectral clustering of single cells using Siamese nerual network combined with improved affinity matrix. Briefings in Bioinformatics, 23(3) (2022).
[5] Kiselev, V.Y., Andrews, T.S., Hemberg, M.: Challenges in unsupervised clustering of single-cell RNA-seq data. Nature Reviews Genetics 20(5), 273–282 (2019)
[6] Li, Y., Rahman, T., Ma, T., Tang, L., Tseng, G.C.: A sparse negative binomial mixture model for clustering RNA-seq count data. Biostatistics (2021).
[7] Mi, X., Bekerman, W., Sims, P.A., Canoll, P.D., Hu, J.: RZiMM-scRNA: A regularized zero-inﬂated mixture model framework for single-cell RNA-seq data. arXiv preprint arXiv:2110.12964 (2021).
[8] Sun, Z., Chen, L., Xin, H., Jiang, Y., Huang, Q., Cillo, A.R., Tabib, T., Kolls, J.K., Bruno, T.C., Lafyatis, R., et al.: A bayesian mixture model for clustering droplet-based single-cell transcriptomic data from population studies. Nature Communications 10(1), 1–10 (2019).
[9] Stassen, S.V., Siu, D.M., Lee, K.C., Ho, J.W., So, H.K., Tsia, K.K.: Parc: ultrafast and accurate clustering of phenotypic data of millions of single cells. Bioinformatics 36(9), 2778–2786 (2020).
[10] Zhang, Y., Zhang, F., Wang, Z., Wu, S., & Tian, W.: scMAGIC: accurately annotating single cells using two rounds of reference-based classification. Nucleic Acids Research, 50(8), e43-e43. (2022).

**Second round of review**

**Reviewer 1**

Many questions have been addressed in this revised version, e.g., adding more experiments of using a larger number of K. However, I have the following concerns for the new results that need to be addressed.


1. The major novelty of the proposed method in comparison with the existing method snbClust is that the proposed model introduces the mixture ZINB model to address the dropout issues in scRNAseq, while snbClust uses the mixture NB model. The basic assumption that it is better to use ZINB to model the dropout effect. As a result, it is particularly critical to justify the replacement of NB with ZINB, and show when ZINB will be superior. For example, we may expect that NB may be the same good, if not better, when dropout rate is very low or 0; while ZINB will outperform for dropout rate is (very) high. The authors conduct experiments on three settings with different dropout levels, but I see quite a few issues that need to clarify:
a. The dropout rates are marked as three levels, but the exact dropout rate values for three different settings are not given. It is not clear how low does the 'low' mean. Please clarify.
b. The performances are perplexing. For the two simulation settings with the same parameters except for the dropout rate, we expect the higher the dropout rate, the worse the clustering performance. However, in Fig S2.a, the ARI value does not follow this pattern except in the setting K=5. Please discuss and explain why.
c. Relevant to (b), the actual performance may also depend on some actual running settings, especially when the methods may not function properly due to the unstableness of the optimization process. For example, we observed that snbClust failed to converge when the dropout rates were too high, and it probably needs more runs with different starting seeds. In our opinion, it may be a fairer comparison when all methods converge.
d. Relevant to (b) and (c), it is good to compare the performance of the proposed model vs snbClust in other simulation settings with dropout rate is low (close or even equal to 0).

2. The proposed model can handle batch effect, while other benchmarks cannot correct batch effect. For fair performance evaluation, the authors should conduct experiments on a simulation without batch effect or apply some batch correction method (e.g., Harmony [1]) before clustering with other benchmarks (e.g., SC3). Such experiments will also help to delineate the contributions of batch effect correction vs the ZINB.

3. The authors have added several simulations for the larger number of clusters. However, they also increase the total number of samples. (300 samples for three clusters, 750 samples for four clusters, and 1500 samples for 10 clusters). The authors should conduct simulations with a fixed total number of samples with a different number of clusters.

4. In the top right figure of Fig.2.a, the performance for the benchmark SC3 seems to decrease as the cluster difference increases, which is perplexing. It is good to provide an explanation and/or double-check the running settings.

5. The authors repeat the experiment many times. It is better to demonstrate the variance of performance in addition to just showing the median value, e.g., using the scatterplot or boxplot. The authors should also conduct a statistical test to prove the superior performance of their proposed model is significant, in comparison with competing methods.


[1] Korsunsky I, Millard N, Fan J, Slowikowski K, Zhang F, Wei K, Baglaenko Y, Brenner M, Loh P-r, Raychaudhuri S. Fast, sensitive and accurate integration of single-cell data with Harmony. Nature Methods; 2019

**Reviewer 2**

|  | The authors have sufficiently addressed my concerns. |
| --- | --- |

The authors have included a dataset containing 5,000 cells. However, from the running time analysis, the proposed method is not for clustering tens of thousands of cells, not to mention millions of cells. Nonetheless, the accuracy of the methods is higher compared with other methods on small to intermediate datasets (<5000 cells).

**Authors Response**

**Point-by-point responses to the reviewers’ comments:**

Response to Editor
We would like to thank you, the associate editor, and two reviewers for your careful review and insightful comments. Following your suggestions, we have conducted another round of careful revision, where major changes have been highlighted in the manuscript. Please see below for our point-by-point response.

1. In particular you should address the remaining comments from Reviewer 1 regarding additional simulations needed to demonstrate the advance of the method
Response: Following the comments from Reviewer 1, we have added the simulation scenarios with a fixed sample size and various numbers of clusters, without dropouts and/or batch effects, and with the dropout rate independent of the cell clusters. Moreover, we have conducted batch correction for SC3 and Seurat using the Harmony approach to provide additional evaluations. Relevant results and discussions have been added to the main text and Supplementary Materials. There is no major change in the main findings.

2. Please also address their concern about the performance of SC3 and add statistical tests where appropriate.
Response: Explanations on the performance of SC3 have been provided. Moreover, in all simulation studies, we have added statistical tests to support the superiority of the proposed method. Relevant results and discussions have been added to the main text and Supplementary Materials.

Response to Reviewer 1
We would like to thank you, the associate editor, and the other reviewer for your careful review and insightful comments. Following your suggestions, we have conducted another round of careful revision, where major changes have been highlighted in the manuscript. Please see below for our point-by-point response.

1.a. The dropout rates are marked as three levels, but the exact dropout rate values for three different settings are not given. It is not clear how low does the 'low' mean. Please clarify.
Response: In simulation, we have considered three levels of dropout rates, including about 5% (low), 45% (medium), and 75% (high), based on the zero-inflated parameters \pi_{jk}'s, which are generated from a Uniform distribution with different parameters. Discussions have been added in Section “Testing ZINBMM using simulated data”, and more detailed settings have been described in Section “Methods”.

1.b. The performances are perplexing. For the two simulation settings with the same parameters except for the dropout rate, we expect the higher the dropout rate, the worse the clustering performance. However, in Fig S2.a, the ARI value does not follow this pattern except in the setting K=5. Please discuss and explain why.
Response: In our study, following recent publications such as Miao et al. (2018) and Cui and Wang (2021), we have simulated data under the setting with dropout rates varying across clusters. Under this setting, as pointed out in Qiu (2020), the dropouts can also serve as informative signals for cell clustering, as the distributions of those dropouts may differ across cell clusters. Thus, compared to the scenarios with a low dropout rate (which have more significantly different biological information across cell types) and the scenarios with a high dropout rate (which have more significantly different dropout distributions across cell types), under the scenarios with a moderate dropout rate (which have weak differences in both biological information and dropout distribution across cell types), all methods show a slightly worse clustering performance. Similar statements that dropouts are informative have also been presented in Li et al. (2019) and Li et al. (2022).
To get more intuition, we have added another setting, under which the dropout rate is independent of the cell types and thus the dropouts are non-informative for cell clustering, and observed that the ARI values decrease as the dropout rate increases.
Discussions and results have been added in Section "Testing ZINBMM using simulated data", Section “Method”, and Supplementary Table S13 of Additional file 2.

1.c. Relevant to (b), the actual performance may also depend on some actual running settings, especially when the methods may not function properly due to the unstableness of the optimization process. For example, we observed that snbClust failed to converge when the dropout rates were too high, and it probably needs more runs with different starting seeds. In our opinion, it may be a fairer comparison when all methods converge.
Response: In our numerical studies, all competing methods have been realized using R packages or codes developed by the corresponding published studies. We acknowledge that performance of these methods may also depend on running settings. We have adopted the running settings recommended by these packages and corresponding studies. Following your suggestion, we have checked convergence of the proposed method as well as all the competing methods, and confirmed that all methods reach convergence specified by the corresponding packages/codes in both simulation and data analysis. Discussions have been added in Section “Method”.

1.d. Relevant to (b) and (c), it is good to compare the performance of the proposed model vs snbClust in other simulation settings with dropout rate is low (close or even equal to 0).
Response: In our analysis, in addition to the simulated datasets generated under the ZINB mixture model, we have also conducted simulations when the data generation model is misspecified. In particular, we have considered the negative binomial mixture model without dropouts and batch effect adopted in snbClust. Relevant results and discussions have been provided in Section “Testing ZINBMM using simulated data” and Table 1. It is observed that the proposed method has a slightly smaller median ARI value than snbClust (0.564 of the proposed method compared to 0.627 of subClust, with P-value computed from the Wilcoxon test being 0.946). However, the proposed method still outperforms the other competing ones.

2. The proposed model can handle batch effect, while other benchmarks cannot correct batch effect. For fair performance evaluation, the authors should conduct experiments on a simulation without batch effect or apply some batch correction method (e.g., Harmony [1]) before clustering with other benchmarks (e.g., SC3). Such experiments will also help to delineate the contributions of batch effect correction vs the ZINB.
Response: We have considered simulated data under the negative binomial mixture model adopted in snbClust and the mixture zero-inflated Poisson model adopted in CIDR. Both models are without batch effects. Under both models, the proposed method has been observed to have satisfactory performance. Relevant results and discussions have been provided in Section “Testing ZINBMM using simulated data” and Table 1.
In addition, following your suggestion, we have conducted batch correction using the Harmony approach for some competing methods, including SC3 and Seurat, and reported the results in Supplementary Table S19 of Additional file 2. We would like to note that among the eleven competing methods, MNN-Graph, MNN-Kmeans, and RZiMM are already able to accommodate batch effects. In addition, since the batch correction step (such as Harmony) usually involves data transformation and/or dimension reduction, sparseKmeans, M3Drop, and NBDrop cannot be combined with a prior batch correction for gene selection. It is also not feasible to introduce a batch correction step for CIDR, scDeepCluster, and snbClust which rely on the raw count or CPM data structure, as the corrected data are usually continuous. The proposed method has been observed to be still superior than SC3 and Seurat incorporating the batch correction. Relevant discussions have been added in Section “Testing ZINBMM using simulated data”.

3. The authors have added several simulations for the larger number of clusters. However, they also increase the total number of samples. (300 samples for three clusters, 750 samples for four clusters, and 1500 samples for 10 clusters). The authors should conduct simulations with a fixed total number of samples with a different number of clusters.
Response: Following your suggestions, we have added simulations with a fixed total sample size of 600 and various numbers of clusters K=3,4,5,6, and 8. Results have been added in Supplementary Table S18 of Additional file 2. When the number of clusters K is 3, 4, or 5, it is observed that the proposed approach remains superior in both clustering accuracy and gene selection. When K grows, the proposed method requires more samples to ensure satisfactory performance, because there are more unknown parameters to be estimated. As a result, clustering performance is slightly worse than SC3 under the setting with K=6 and 8. However, such performance can be easily enhanced by a larger sample size. The current simulation studies show that 150 samples per cluster can already provide satisfactory results, which can be easily satisfied with practical data. For example, the majority of cell types for the five real datasets examined in our study have sizes greater than 150. Relevant discussions have been added in Section “Testing ZINBMM using simulated data”.

4. In the top right figure of Fig.2.a, the performance for the benchmark SC3 seems to decrease as the cluster difference increases, which is perplexing. It is good to provide an explanation and/or double-check the running settings.
Response: Following your suggestion, we have carefully checked the running settings. We conjecture that this phenomenon is potentially attributed to the following reasons. On one hand, in simulation, we randomly selected 1,000 values from the baseline expression levels in the Zeisel dataset. Along with the dropout scheme and negative binomial generation model, there are multiple levels of randomness. In addition, both dropout distributions and biological cluster differences contribute to clustering. Thus, when the dropout rate is higher (top right figure of Figure. 2a), together with multiple levels of randomness, the results do not significantly depend on the biological differences between clusters. On the other hand, the median ARI values for SC3 in the top right figure of Figure. 2a are all close to 1, which does not leave much space for clustering performance to gain significant differences. Discussions have been added in Section “Testing ZINBMM using simulated data”.

5. The authors repeat the experiment many times. It is better to demonstrate the variance of performance in addition to just showing the median value, e.g., using the scatterplot or boxplot. The authors should also conduct a statistical test to prove the superior performance of their proposed model is significant, in comparison with competing methods.
Response: We would like to note that, with limited space, we have only reported the median values of measures using scatter plots to better present comprehensive information under various scenarios in one figure. Following your suggestions, we have provided the results of medians, median absolute deviations (MADs), and P-values from statistical testing in Additional ﬁle 2: Tables S1-S19. Overall, the proposed method can achieve superior performance in both clustering and gene selection, and the significance of improvement is supported by statistical testing. Discussions have been added in Section “Testing ZINBMM using simulated data”.

Reference
[1] Cui, T., Wang, T.: Joint for large-scale single-cell RNA-sequencing analysis via soft-clustering and parallel computing. BMC Genomics 22, 1–16 (2021)
[2] Li, R., Quon, G.: scbfa: modeling detection patterns to mitigate technical noise in large-scale single-cell genomics data. Genome Biology 20, 1–20 (2019)
[3] Li, H., Li, H., Zhou, J., Gao, X.: Sd2: spatially resolved transcriptomics deconvolution through integration of dropout and spatial information. Bioinformatics 38(21), 4878–4884 (2022)
[4] Miao, Z., Deng, K., Wang, X., Zhang, X.: Desingle for detecting three types of differential expression in single-cell RNA-seq data. Bioinformatics 34(18), 3223–3224 (2018)
[5] Qiu, P.: Embracing the dropouts in single-cell RNA-seq analysis. Nature Communications 11(1), 1–9 (2020)

Response to Reviewer 2
We would like to thank you, the associate editor, and the other reviewer for your careful review and insightful comments. Following your suggestions, we have conducted another round of careful revision, where major changes have been highlighted in the manuscript. Please see below for our point-by-point response.

1. The authors have included a dataset containing 5,000 cells. However, from the running time analysis, the proposed method is not for clustering tens of thousands of cells, not to mention millions of cells. Nonetheless, the accuracy of the methods is higher compared with other methods on small to intermediate datasets (<5000 cells).
Response: We thank you for your comments. We have further applied the proposed method to a simulated dataset with 100,000 cells. The proposed analysis takes about 9.8 hours on a laptop with an Intel Core i5 processor and 16 GB RAM. We conjecture that the proposed analysis can be applicable to large datasets. Discussions have been added in Section “Testing ZINBMM using simulated data”.

**Third round of review**

**Reviewer 1**

This paper proposes a mixture ZINB model ZINBMM to simultaneously implement clustering and gene selection for scRNA-seq data. ZINBMM is able to handle batch effects and dropout in the clustering process. The authors have conducted several additional experiments to demonstrate the performance associated with various sample sizes, the number of clusters, and the impact of dropout. However, the following concerns remain:

1 Novelty issue. The authors mentioned that “RZiMM relies on continuously normalized values, which may lead to a potential loss of information.” However, apart from the zero-inflated mixture Gaussian distribution, RZiMM also employs a zero-inflated mixture negative binomial distribution, called RZiMM-NB, to model scRNA-seq data. Both RZiMM-NB and the proposed model consider the dropouts and the batch effect. Thus, compared to RZiMM-NB, I feel the novelty and the contributions are incremental. The advantages of the proposed method are not clear, in comparison with RZiMM-NB.

2. Quite a few problems in optimization. (a)Lines 49-50, Page 20, the objective functions for optimization are not given. Please provide. Are they convex, since the numeric optimization Newton-Raphson method is used? (b) It is not clear whether the parameters output from the M-step including the two parameters concerned in (a) are maximum likelihood estimates. If not, what is its implication in the EM algorithm? e.g., will it invalidate the nice properties of the EM algorithm? (c) the problem of identifiability. The ZINB model is a mixture, then based on it the mixture of ZINB has another layer of mixture. Can the authors show if mixtures of ZINB are identifiable? If not, what would be its implication in the EM algorithm? (d) How the candidate values of λ are set is not clear, which seems critical for balancing the running time and the chance of not missing optimal solutions.

3. Issues about dropout. (a) The authors have conducted an ablation study (Table S13) under which “the dropout rate is independent of the cell types and thus the dropouts are non-informative for cell clustering, and observe that the ARI values decrease as the dropout rate increases.” Moreover, the authors also mention that “the dropouts can also serve as informative signals for cell clustering, as the distributions of those dropouts may differ across cell clusters” and other experiments are “under the settings with dropout rates varying across clusters.” However, in the Section Data simulation, the authors state that the dropout rate for gene j in cluster k, \pi_{jk}, is sampled from the same uniform distribution, e.g., U(0, 0.3) for the low dropout rate setting. This dropout setting is not dependent on the cluster k. Please clarify the statement or the simulation settings.

3(b) Relevant to 3(a), under the simulation setting where dropout rates vary across clusters, it is expected that, for a given biological cluster difference, an increase in the dropout rate should correspond to an increase in clustering performance, since a large dropout rate can introduce more information. However, as illustrated in the top three figures in Fig 2.a, for a fixed biological cluster difference (either Low, Medium or High), the ARI of the proposed method under different levels of dropout rate reveals that the medium dropout rate results in the worst performance (e.g., the red points in the first column of the top three figures in Fig 2.a). Please explain why.

3(c) Relevant to 3(b), the authors need to explain the statement “Thus, compared to the scenarios with a low dropout rate (which have more significantly different biological information across cell types) and the scenarios with a high dropout rate (which have more significantly different dropout distributions across cell types), under the scenarios with a moderate dropout rate (which have weak differences in both biological information and dropout distribution across cell types), all methods show a slightly worse clustering performance.”

**Authors Response**

**Point-by-point responses to the reviewers’ comments:**

Response to Reviewer 1
We would like to thank you and the editors for your careful review and insightful comments. Following your suggestions, we have conducted another round of careful revision, where major changes have been highlighted in the manuscript. Please see below for our point-by-point response.

1 Novelty issue. The authors mentioned that “RZiMM relies on continuously normalized values, which may lead to a potential loss of information.” However, apart from the zero-inflated mixture Gaussian distribution, RZiMM also employs a zero-inflated mixture negative binomial distribution, called RZiMM-NB, to model scRNA-seq data. Both RZiMM-NB and the proposed model consider the dropouts and the batch effect. Thus, compared to RZiMM-NB, I feel the novelty and the contributions are incremental. The advantages of the proposed method are not clear, in comparison with RZiMM-NB.
Response: As the objective function, algorithm, and code for RZiMM-NB were not available in Mi et al., 2021 when we submitted the manuscript, we considered RZiMM based on the Gaussian distribution instead in the early version of our article. With the updated R codes in https://github.com/SkadiEye/RZiMM, we have now conducted the analysis using RZiMM-NB (which is still referred to as RZiMM in the article) and updated the results. It is observed that the performance of RZiMM-NB is slightly better than that of RZiMM but inferior to the proposed method.
The proposed model advances from the RZiMM model in the following aspects:
（1）First, RZiMM considers one zero-inflated model in the form of
(1-π_j )I(X_ij=0)+π_j I(X_{ij}≠0)⋅G(X_{ij}∣μ_{ij},σ_j^2 ),
with the mean value μ_{ij}=e_(j,k(i) )+∑_(p=1)^P G_{ip} m_{jp} depending on the specific subgroup. Here, G_{ip}’s are the binary subgroup indicators. Thus, RZiMM can only achieve hard clustering with the estimated binary G_{ip}. Significantly different from RZiMM, we have developed a ZINB mixture model with a probability mass function
∑_(k=1)^K  p_k⋅[π_{jk} I(X_{ij}=0)+(1-π_{jk}) f_NB (X_{ij};μ_{ijk},ϕ_j)],
where p_k’s are the mixing proportions. Different from RZiMM, the proposed method introduces the mixing proportions to accommodate cell heterogeneity and can achieve soft clustering. The mixture model has had great success in biological heterogeneity analysis studies and has the advantage of more meaningful probabilistic interpretations.
（2） Second, for the dropout event, RZiMM simply models all zeros with one component (1-π_j )I(X_ij=0), which assumes that all zeros are generated by technical noises and ignores the differences between dropout zeros and expression zeros, leading to additional bias. Significantly different from RZiMM, the proposed method models zeros with two components π_jk I(X_ij=0) and (1-π_jk ) f_NB (X_ij;μ_ijk,ϕ_j ), which can effectively accommodate both technical and biological zeros, leading to more accurate estimation and identification results.
（3） Third, RZiMM introduces the pairwise penalty and gene importance scoring to quantify the importance of genes. This requires another threshold, which cannot be determined automatically and may involve some subjectivity. Significantly different from RZiMM, we have introduced the penalty
to automatically identify cell type-specific genes, where β_jk’s are promoted to shrink to the global value β_j^* and the important genes are those with at least one β_jk different from β_j^*.
Discussions have been added in the Section “Background”, where the novelty and contributions of the proposed method have been clearly explained.

2. (a). Lines 49-50, Page 20, the objective functions for optimization are not given. Please provide. Are they convex, since the numeric optimization Newton-Raphson method is used?
Response: The objective functions of optimization have been added in Section “Methods” and are in the form of the weighted loglikelihood of a negative binomial distribution and are nonconcave. We adopt the BFGS quasi-Newton method for optimizing parameters \phi and \gamma. This quasi-Newton algorithm has fewer constraints on the convexity of the target function. The BFGS quasi-Newton method has been widely adopted and shown to have satisfactory performance in published studies such as Lim et al. (2021), Akram et al. (2022), and Jiang et al. (2023). We have followed these studies and used the R function ‘optim’ to realize the BFGS quasi-Newton method. Discussions have been added in the Section “Methods”.

2. (b). It is not clear whether the parameters output from the M-step including the two parameters concerned in (a) are maximum likelihood estimates. If not, what is its implication in the EM algorithm? e.g., will it invalidate the nice properties of the EM algorithm?
Response: We would like to note that, for optimization with mixture models, the EM algorithm has been very popularly adopted with latent variables introduced and the log-likelihood function rewritten into the complete-data objective function. The EM algorithm can guarantee the stability of estimation under fairly mild conditions. We refer to Wu (1983) and Nanjundan (2006) for more discussions. Furthermore, when multiple parameters are involved, for those whose closed-form optimizers are not available, iterative approaches such as the Newton-Raphson or quasi-Newton methods have been commonly adopted, and satisfactory performance has been broadly observed – see for example Cai and Wang (2014), Lim et al. (2022), and Jiang et al. (2023). Following published studies, we have also adopted the BFGS quasi-Newton method for optimization of the dispersion and batch effect parameters and observed satisfactory numerical performance. Discussions have been added in the Section “Methods”.

2. (c). the problem of identifiability. The ZINB model is a mixture, then based on it the mixture of ZINB has another layer of mixture. Can the authors show if mixtures of ZINB are identifiable? If not, what would be its implication in the EM algorithm?
Response: The identifiability of mixture distributions is unfortunately still a wide-open problem (McLachlan et al., 2019, Ranalli and Rocci, 2023). In Yakowitz and Spragins (1968), finite mixtures of negative binomial distributions have been shown to be identifiable. The identifiability of the zero-inflated model has been established in Li (2012). For one simulated replicate, we have conducted the proposed analysis multiple times and obtained the same estimator, which may suggest the identifiability of the proposed method.
Moreover, the two-layer mixture model has been common in recent studies – see for example Wang and Xiang (2017), Murali (2020), and Wu and Ma (2018). In these studies, a two-layer EM algorithm has been adopted with two separate sets of latent variables introduced in the optimization procedure.
We have followed these studies and adopted the two-layer EM algorithm. The numerical investigation has suggested the identifiability of the proposed method. In this study, we have mostly focused on methodological development and implementation. Theoretical studies on identiﬁability may be beyond the scope of this study. Discussions have been added in the Section “Discussions”.

2.(d). How the candidate values of λ are set is not clear, which seems critical for balancing the running time and the chance of not missing optimal solutions.
Response: In our numerical studies, we have considered a candidate sequence of λ by setting the minimum and maximum values as 0.01 and 20 and constructing an equally spaced sequence within the given range with the sequence length being 10. As in perhaps all studies, the choice of the candidate tuning parameters cannot be fully objective. The adopted strategy has been very common in published studies (Lim et al., 2021, Mi et al., 2021). Such a choice of candidate values has led to satisfactory performance in our numerical studies. In data analysis, a more refined search can be obtained by expanding the range of candidate parameters, and a reduced range of candidates can help improve the computation efficiency. Discussions have been added in the Section “Methods”.

3.(a). Issues about dropout. The authors have conducted an ablation study (Table S13) under which “the dropout rate is independent of the cell types and thus the dropouts are non-informative for cell clustering, and observe that the ARI values decrease as the dropout rate increases.” Moreover, the authors also mention that “the dropouts can also serve as informative signals for cell clustering, as the distributions of those dropouts may differ across cell clusters” and other experiments are “under the settings with dropout rates varying across clusters.” However, in the Section Data simulation, the authors state that the dropout rate for gene j in cluster k, \pi_{jk}, is sampled from the same uniform distribution, e.g., U(0, 0.3) for the low dropout rate setting. This dropout setting is not dependent on the cluster k. Please clarify the statement or the simulation settings.
Response: Under the settings where the dropout rate depends on the clusters, although the same uniform distribution has been considered, we have sampled K different π_jk for k=1,…,K from this distribution. Thus, π_jk≠π_(jk^' ) when k≠k^'. Under the settings where the dropout rate does not depend on the clusters, we have sampled only one π_j from the uniform distribution, and set π_jk= π_j for all k=1,…,K. We have revised the relevant descriptions in Section “Data simulation”.

3.(b). Relevant to 3(a), under the simulation setting where dropout rates vary across clusters, it is expected that, for a given biological cluster difference, an increase in the dropout rate should correspond to an increase in clustering performance, since a large dropout rate can introduce more information. However, as illustrated in the top three figures in Fig 2.a, for a fixed biological cluster difference (either Low, Medium or High), the ARI of the proposed method under different levels of dropout rate reveals that the medium dropout rate results in the worst performance (e.g., the red points in the first column of the top three figures in Fig 2.a). Please explain why.
Response: In our simulation studies, for each gene, its expression is generated from the NB distribution with probability 1-\pi_{jk} and is directly set to zero with probability \pi_{jk}. Thus, although the biological cluster difference is fixed (where the considered NB distribution is the same), the signals of these differentially expressed genes vary across different dropout rates, as some nonzero counts generated from the NB distribution may be replaced by zeros due to dropout. As a result, the performance of the methods depends not only on the dropout distribution but also on the underlying signals of the differentially expressed genes.
To get more intuitive insight, we consider three replicates simulated from the scenarios with three different levels of dropout rate but with the same biological cluster difference and batch effect. A special replicate with no dropouts is also considered. For these four replicates, the heatmaps of the 50 biologically important genes (with \beta_{jk}'s varying across clusters) and 20 none important genes and the boxplots of the mean expressions of these important genes and the proportions of zero observations in the three clusters are presented in Fig. S2 (Additional file 1).
It is observed in Fig. S2 (b) that, compared to the scenarios with a low and high dropout rate, the diversity of expression distributions is less significant under the scenario with a medium dropout rate, leading to the inferior clustering performance of the methods. Specifically,
（1）Under the scenario with a low dropout rate, the diversity of expression distributions across clusters (p-value 0.00027) is mainly due to the biological cluster difference, which is not covered by the limited number of zeros (see Fig. S2 (a)).
（2）Under the scenario with a medium or high dropout rate, biological information is somehow covered and confounded by dropout zeros (Fig. S2 (a)).
（3）When the dropout rate is high, since most of the expressed values are zeros, the differences across clusters are dominated by the distribution differences of zero observations across clusters, which are significant (p value 0.008) as shown in Fig. S2 (c), leading to significantly different mean expressions (p value 0.0071 in Fig. S2 (b)).
（4）When the dropout rate is medium, both the differences of biological information (covered by dropout zeros) and dropout distribution (not strong enough to be distinctive with a p value of 0.65) across clusters are weak, leading to slightly worse clustering performance.
Discussions have been added in the Section “Testing ZINBMM using simulated data”.

3.(c) Relevant to 3(b), the authors need to explain the statement “Thus, compared to the scenarios with a low dropout rate (which have more significantly different biological information across cell types) and the scenarios with a high dropout rate (which have more significantly different dropout distributions across cell types), under the scenarios with a moderate dropout rate (which have weak differences in both biological information and dropout distribution across cell types), all methods show a slightly worse clustering performance.”
Response: We refer to the response to 3(b). Discussions have been added to the main text and Supplementary Materials.

Reference
[1] Akram, M. N., Abonazel, M. R., Amin, M., Kibria, B. G., & Afzal, N. (2022). A new Stein estimator for the zero‐inflated negative binomial regression model. Concurrency and Computation: Practice and Experience, 34(19), e7045.
[2] Cai, Z., & Wang, X. (2014). Selection of mixed copula model via penalized likelihood. Journal of the American Statistical Association, 109(506), 788-801.
[3] Jiang, R., Zhan, X., & Wang, T. (2023). A flexible zero-inflated Poisson-Gamma model with application to microbiome sequence count data. Journal of the American Statistical Association, 118(542), 792-804.
[4] Lim, D. K., Rashid, N. U., & Ibrahim, J. G. (2021). Model-based feature selection and clustering of RNA-seq data for unsupervised subtype discovery. The Annals of Applied Statistics, 15(1), 481-508.
[5] Li, C. (2012). Identifiability of zero-inflated Poisson models. Brazilian Journal of Probability and Statistics, 26 (3), 306-312.
[6] Mi, X., Bekerman, W., Sims, P. A., Canoll, P. D., & Hu, J. (2021). RZiMM-scRNA: A regularized zero-inflated mixture model framework for single-cell RNA-seq data. arXiv preprint arXiv:2110.12964.
[7] Murali, R. (2020, December). Analysis of COVID-19 in different countries using Wigner energy distribution and clustering. In 2020 19th IEEE international conference on machine learning and applications (ICMLA), 592-597.
[8] McLachlan, G. J., Lee, S. X., & Rathnayake, S. I. (2019). Finite mixture models. Annual Review of Statistics and its Application, 6, 355-378.
[9] Nanjundan, G. (2006). An EM algorithmic approach to maximum likelihood estimation in a mixture model. Vignana Bharathi, 18(1), 7-13.
[10] Ranalli, M., & Rocci, R. (2023). Composite likelihood methods for parsimonious model-based clustering of mixed-type data. Advances in Data Analysis and Classification, 1-27.
[11] Wang, S., & Xiang, L. (2017). Two-layer EM algorithm for ALD mixture regression models: A new solution to composite quantile regression. Computational Statistics & Data Analysis, 115, 136-154.
[12] Wu, C. J. (1983). On the convergence properties of the EM algorithm. The Annals of Statistics, 95-103.
[13] Wu, D., & Ma, J. (2018). A two-layer mixture model of Gaussian process functional regressions and its MCMC EM algorithm. IEEE Transactions on Neural Networks and Learning Systems, 29(10), 4894-4904.
[14] Yakowitz, S. J., & Spragins, J. D. (1968). On the identifiability of finite mixtures. The Annals of Mathematical Statistics, 39(1), 209-214.

**Fourth round of review**

**Reviewer 1**

All previous comments have been addressed.
